# A deep learning framework for modeling structural features of RNA-binding protein targets

**DOI:** 10.1093/nar/gkv1025

**Published:** 2015-10-13

**Authors:** Sai Zhang, Jingtian Zhou, Hailin Hu, Haipeng Gong, Ligong Chen, Chao Cheng, Jianyang Zeng

**Affiliations:** 1 Institute for Interdisciplinary Information Sciences, Tsinghua University, Beijing 100084, China; 2 Department of Pharmacology and Pharmaceutical Sciences, School of Medicine, Tsinghua University, Beijing 100084, China; 3 School of Life Sciences, Tsinghua University, Beijing 100084, China; 4 MOE Key Laboratory of Bioinformatics, Tsinghua University, Beijing 100084, China; 5 Department of Genetics, Institute for Quantitative Biomedical Sciences, Norris Cotton Cancer Center, Geisel School of Medicine at Dartmouth, Hanover, NH 03755, USA

## Abstract

RNA-binding proteins (RBPs) play important roles in the post-transcriptional control of RNAs. Identifying RBP binding sites and characterizing RBP binding preferences are key steps toward understanding the basic mechanisms of the post-transcriptional gene regulation. Though numerous computational methods have been developed for modeling RBP binding preferences, discovering a complete structural representation of the RBP targets by integrating their available structural features in all three dimensions is still a challenging task. In this paper, we develop a general and flexible deep learning framework for modeling structural binding preferences and predicting binding sites of RBPs, which takes (predicted) RNA tertiary structural information into account *for the first time*. Our framework constructs a unified representation that characterizes the structural specificities of RBP targets in all three dimensions, which can be further used to predict novel candidate binding sites and discover potential binding motifs. Through testing on the real CLIP-seq datasets, we have demonstrated that our deep learning framework can automatically extract effective hidden structural features from the encoded raw sequence and structural profiles, and predict accurate RBP binding sites. In addition, we have conducted the *first* study to show that integrating the additional RNA tertiary structural features can improve the model performance in predicting RBP binding sites, especially for the polypyrimidine tract-binding protein (PTB), which also provides a new evidence to support the view that RBPs may own specific tertiary structural binding preferences. In particular, the tests on the internal ribosome entry site (IRES) segments yield satisfiable results with experimental support from the literature and further demonstrate the necessity of incorporating RNA tertiary structural information into the prediction model. The source code of our approach can be found in https://github.com/thucombio/deepnet-rbp.

## INTRODUCTION

RNA-binding proteins (RBPs) play important roles in various cellular processes, such as alternative splicing, RNA editing, mRNA localization and translational regulation (1). RBPs contain several special RNA-binding domains (RBDs), e.g. the RNA recognition motif (RRM) and the hnRNP K-homology (KH) domains, which recognize their target sites related to the RNA primary sequence and the corresponding structural profiles (2). Although it has been shown that several important diseases, such as neurodegenerative disorders, cancers and cardiovascular diseases, can be caused by the dysfunctions of certain RBPs (3,4), relatively few RBPs have been well characterized. Therefore, identifying RNA–protein interactions and modeling RBP binding preferences are important for decoding the post-transcriptional processes involving RBPs and their mechanisms of pathogenesis in human diseases.

Recently, the advent of high-throughput experimental methods, such as the cross-linking immunoprecipitation coupled with high-throughput sequencing (CLIP-seq) protocols, has greatly advanced the genome-wide studies of RNA–protein interactions (5–8). Despite the success stories of these experimental techniques, the collected data still suffer from the false-positive and false-negative problems due to the experimental noises and current limitations in these techniques (e.g. the limited mappability of splice sites). To address these issues and identify missing RBP binding sites, numerous computational methods (9–13) have been proposed to complete the RNA–protein interactome. In principle, the collected CLIP-based data can be used as training data and fed into certain computational predictors to detect new RBP targets.

Although it is well known that the structural factors, such as RNA secondary structure, can significantly influence the RBP binding behaviors (2), most computational models (11,13) only consider the primary sequence and secondary structural features, and assume that the structural profiles of different nucleotide positions are independent with each other. With such assumptions, these models have limited performance in predicting RBP binding sites (e.g. in those base-pairing regions). A novel computational method called GraphProt (10) with predefined secondary structural features has been recently proposed to address this issue. Although existing computational approaches can accurately predict RBP binding sites to some extent (10–11,13), most of them lack the flexibility to integrate more structural information. Also, few of them are particularly designed to exploit the tertiary structural features to investigate the impacts of RNA tertiary structure on RBP binding. On the other hand, despite the paucity of the high-resolution structural data of RNA–protein complexes, the current available databases of RNA 3D motifs can still offer a new and useful source of information for studying the tertiary structural binding preferences of RBPs. Thus, this leaves a certain gap to fully understand the impacts of the integrated RNA structural features on RNA–protein interactions. To tackle this issue, we need to develop an effective method to encode both RNA secondary and tertiary structures in addition to the base sequence, as well as a general machine learning framework to integrate the encoded RNA sequence and structural profiles to model RBP binding preferences and detect new target sites.

Nowadays, in the machine learning field, deep learning (14,15) has become a popular and powerful tool for analyzing complicated data and capturing hierarchical representations of the intrinsic latent features in data. A number of empirical studies have demonstrated that deep learning can achieve the state-of-the-art prediction performance in various learning tasks, e.g. image classification (15), automatic speech recognition (16) and natural language processing (17). Also, deep learning has been successfully applied to solve several prediction problems in the field of computational biology and bioinformatics, such as protein structure prediction (18), chemoinformatics (19) and RNA splicing prediction (20). In the multimodal scenario, in which the input data consist of multiple modalities (e.g. the audio and visual signals in speech recognition), multimodal deep learning architectures (21–23) have been developed to effectively integrate these modalities and capture common latent features across them for accurate inference and prediction. The power of modeling complicated statistical characteristics and the flexibility of integrative data analysis of deep learning have given rise to a wide range of applications of this new machine learning technique.

In this study, we develop a novel deep learning framework to predict RBP target sites and model their sequence and structural specificities by systematically integrating the RNA primary sequence, (predicted) secondary and tertiary structural features. We have made the following contributions: (i) introduction of a novel method to encode RNA base sequence and secondary structure; (ii) introduction of a new method to construct the RNA tertiary structural profiles; (iii) development of a deep learning model to integrate the RNA sequence, secondary and tertiary structural profiles, and construct a unified representation to extract the hidden structural features of RBP targets; (iv) the *first* study of exploiting the RNA tertiary structural features to predict RBP binding sites and excellent test results on the real CLIP-seq data, in which most of the sequence and structural motifs derived by our algorithm own support from experimental evidence or previous studies in the literature; (v) novel 3D structural binding motifs of RBPs derived by our model, which can provide important clues for understanding the mechanisms of RBPs’ action in the post-transcriptional control of RNAs; (vi) test results on predicting PTB binding sites for the internal ribosome entry site (IRES) segments that agree with the previous experimental studies.

## MATERIALS AND METHODS

### Overview

**Figure 1.**
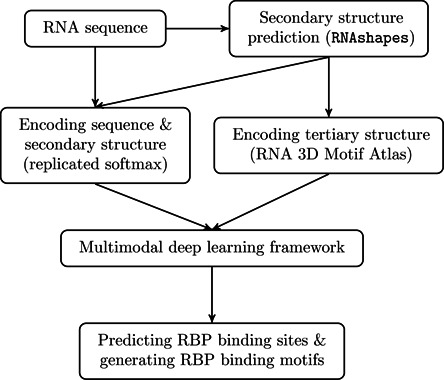
Schematic overview of our deep learning framework.

Figure 1 illustrates the schematic overview of our deep learning framework, which consists of three main phases, including data encoding, training and application phases. In the data encoding phase, we first truncate RNA sequence around the bound site identified by the CLIP-based experiments and use the tool RNAshapes (24) to predict probable secondary structures. Then we encode the primary sequence and secondary structure using the replicated softmax model, a probabilistic graphical model that is originally designed to discover the latent topics of documents in the natural language processing field. To encode RNA tertiary structure, we exploit the tertiary motifs predicted by JAR3D (25,26), a computational framework that infers the probable tertiary structural motifs in the hairpin and internal loop regions based on RNA 3D Motif Atlas (R3DMA) (27). The encoded tertiary structural profiles represent possible tertiary motifs folded by the RNA sequence. In the training phase, we build a multimodal deep belief network (DBN) to integrate all the aforementioned encoded sequence and structural profiles. We use the experimentally identified RBP binding sites derived from the available CLIP-seq datasets to train our multimodal deep learning model. In the application phase, the trained deep architecture is used to detect novel RBP binding sites on the genome that we are interested in, and generate the sequence and structural specificities in target recognition.

### Encoding RNA base sequence and secondary structure

In the literature of RBP target recognition, there is a tendency to integrate RNA sequence and structural profiles as methodic features into the machine learning models to predict unknown RBP binding sites. For example, MEMERIS (12) incorporates the accessibility (i.e. the probability of a nucleotide being unpaired) (28) of the RNA sequences to identify RBP targets. RNAcontext (11) further differentiates various forms of loops, e.g. hairpin, multiloop and bulge, in RNA secondary structure for RBP binding site prediction. In addition, CapR (13) considers the positional correlations of different structural contexts by modeling the joint distribution of the position and type of RNA secondary structure in the target sequences. Furthermore, GraphProt (10) integrates the local structural information (e.g. RNA secondary structural contexts and their dependency) using a graph kernel model to explore the RBP binding preferences. The performance of these methods depends on how accurately and reasonably the RNA sequence and structure are encoded in the computational model. Because of their *explicit* encoding principle, these prediction approaches may miss subtle structural features hidden in the input data, which cannot be captured by the handcrafted feature extraction methods. In addition, they lack the flexibility of integrating new structural information, such as RNA tertiary structure. An alternative of such a ‘complex input simple model’ principle is a relatively complicated model with simple and nonmethodical input (29). In other words, by feeding a sophisticated model with simple but sufficient raw input data, we can let it automatically learn effective hidden features, which yields an *implicit* encoding method. Here, we adopt the latter principle.

#### Restricted Boltzmann machines

We first introduce the restricted Boltzmann machines (RBMs) (30), which are fundamental building blocks of the deep learning architectures (31). Generally speaking, RBMs are Markov random fields that characterize the probability distribution of a set of binary variables. More specifically, an RBM consists of two layers, including a visible layer of observable variables (or referred to as vertices) and a hidden layer of unobservable variables, with the restriction that visible and hidden layers are fully connected and there is no connection between vertices in the same layer. An RBM example is shown in Supplementary Figure S1(A). These structural characteristics make the variables in the same layer conditionally independent with each other, given the variables in the other layer. In our deep learning framework, we use an RBM to integrate the RNA sequence, secondary and tertiary structural profiles, with the implicit assumption that the encoded RNA sequence and structure can be modeled as a joint probability distribution determined by a number of hidden variables. In other words, the input RNA sequence and structural profiles are relevant to each other and can be regarded as a joint effect of these hidden factors. Such a modeling strategy may reflect the real setting of the RBP binding behavior.

Let $w$_*ij*_ denote the real-valued weight associated with the edge between visible variable *V*_*i*_ and hidden variable *H*_*j*_, let *b*_*i*_ and *c*_*j*_ be the bias terms associated with visible variable *V*_*i*_ and hidden variable *H*_*j*_, respectively. We use lowercase letters $v$_*i*_ and *h*_*j*_ to represent the values of the corresponding variables *V*_*i*_ and *H*_*j*_, respectively. We use $\mathbf {W}$, $\mathbf {b}$ and $\mathbf {c}$ to denote the vector representations of the corresponding parameters, respectively, and $\mathbf {v}$ and $\mathbf {h}$ to denote the vector representations of the states of visible and hidden variables, respectively. For an RBM with *m* visible variables and *n* hidden variables, its joint probability density function is defined by

(1) \begin{equation*} p(\mathbf {v},\mathbf {h};\boldsymbol{\theta })=\frac{1}{Z(\boldsymbol{\theta })}\exp (-E(\mathbf {v},\mathbf {h};\boldsymbol{\theta })), \end{equation*}

where $(\mathbf {v},\mathbf {h})\in \lbrace 0,1\rbrace ^{m+n}$, $\boldsymbol{\theta }=(\mathbf {b},\mathbf {c},\mathbf {W})$, $Z(\boldsymbol{\theta })$ is the partition function, and the energy function $E:\lbrace 0,1\rbrace ^{m+n}\rightarrow \mathbb {R}$ is defined by

(2) \begin{equation*} E(\mathbf {v},\mathbf {h};\boldsymbol{\theta })= -\sum _{i=1}^m\sum _{j=1}^n w_{ij} v_i h_j-\sum _{i=1}^m b_i v_i-\sum _{j=1}^n c_j h_j. \end{equation*}

To train the model, we can perform the maximum likelihood estimation (MLE). In particular, the *contrastive divergence* (CD) algorithm (32) is often used to approximate the gradient of the log-likelihood of the training data. With such an approximation, we can update the RBM parameters using the standard gradient ascent strategy.

#### Replicated softmax

The replicated softmax model (33) is a *topic model* that characterizes the word distribution in documents based on the latent topics. An example of a replicated softmax model is shown in Supplementary Figure S1(B). In our context, we treat the RNA sequences (in which each letter stands for the nucleotide type or secondary structure type at the corresponding base position) as ‘documents’, RNA subsequences as ‘words’ and a collection of distinct RNA subsequences as a ‘dictionary’. The ‘latent topics’ in the replicated softmax represent unknown factors that determine the sequence or structural features of RBP binding sites. Hereinafter, we will use *RNA documents*, *RNA words*, *RNA dictionary* and *RNA topics* to denote the corresponding concepts that are similar to those in the natural language processing field.

For a given RNA document $\cal {D}$ of size *D* with an RNA dictionary $\cal {K}$ of size *K*, there exists a matrix representation $\mathbf {V}$ of size *K* × *D*, where $v_i^k=1$ if and only if its *i*th RNA word takes the *k*th value of $\cal {K}$. In replicated softmax, the probability density function of $\mathbf {V}$ is defined by

(3) \begin{equation*} p(\mathbf {V};\boldsymbol{\theta })=\frac{1}{Z(\boldsymbol{\theta })}\sum _{\mathbf {h}}\exp \left(-E(\mathbf {V},\mathbf {h};\boldsymbol{\theta })\right), \end{equation*}

where $E(\mathbf {V},\mathbf {h};\boldsymbol{\theta })$ is defined by

(4) \begin{eqnarray*} E(\mathbf {V},\mathbf {h};\boldsymbol{\theta })&=&-\sum _{i=1}^D\sum _{j=1}^N\sum _{k=1}^K w_{ij}^k v_i^k h_j-\sum _{i=1}^D\sum _{k=1}^K b_i^k v_i^k -D\sum _{j=1}^N c_j h_j\nonumber \\ &=&-\sum _{j=1}^N\sum _{k=1}^K w_j^k \hat{v}^k h_j-\sum _{k=1}^K b^k \hat{v}^k-D\sum _{j=1}^N c_j h_j, \end{eqnarray*}

where *N* stands for the number of hidden variables representing the latent RNA topics, $w_{ij}^k$ equals to each other for fixed *j* and *k* as we treat the RNA words in the RNA document $\cal {D}$ as examples sampled from the replicated softmax model independently, and $\hat{v}^k=\sum _{i=1}^D v_i^k$ denotes the number of the *k*th RNA word of $\cal {K}$ in the RNA document. For a collection of *M* RNA documents $\lbrace \mathbf {V}_m\rbrace _{m=1}^M$, the training criterion is to maximize the average log-likelihood of the training data. Here, the CD algorithm can also be performed to learn the model parameters, including $w$_*ij*_, *b*_*i*_ and *c*_*j*_ (33). A more detailed explanation of the replicated softmax model can be found in Supplementary Section S1.

#### Constructing the RNA primary sequence and secondary structural profiles

**Figure 2.**
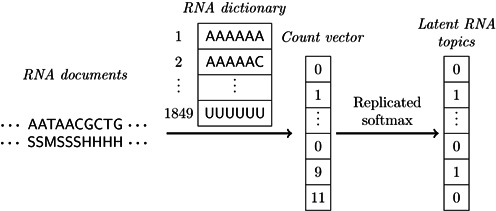
Schematic illustration of encoding RNA primary sequence and secondary structure. The definitions of RNA document, RNA dictionary, RNA topic and count vector are described in the text. Here, we only show the process of encoding the primary sequence. A similar scheme can be conducted to encode RNA secondary structure (see Section ‘Constructing the RNA primary sequence and secondary structural profiles’ for details).

When constructing the base sequence and secondary structural profiles of RBP targets, the *viewpoint* regions are the RBP binding sites identified by the CLIP-seq experiments. When encoding an RNA primary sequence, the four elementary bases (i.e. A, G, C and U) are first used to construct the RNA dictionary, which includes all possible subsequences (i.e. RNA words) of fixed length *k* that are composed of bases in different orders. These subsequences of length *k* are also referred to as *k*-mers. In principle, an RNA dictionary with longer RNA words can provide a more accurate description of the RNA sequence. On the other hand, it is usually time- and space-consuming to process relatively long RNA words, since both time and space complexities grow exponentially as the length of RNA words increases. In our framework, the length of RNA words is set to be six, i.e. *k* = 6. Our additional tests (see Supplementary Section S5) have shown that slightly different RNA word length does not impact much on the model performance. We then use a sliding window of size 6 and step size 1 to scan the viewpoint region for each RNA document. After that, we construct a vector, called *count vector*, to record the occurrence of each RNA word in the scanning process. In particular, the *i*th element in the count vector records the number of times that the *i*th word in the RNA dictionary appears during the scanning process. This count vector is then used as the input fed into the replicated softmax model, as described in the previous section, to extract the corresponding latent RNA topics of the base sequence (see Figure 2).

The method of encoding RNA secondary structure is almost the same as that of encoding the primary sequence, except with the following processes. First, since the experimental secondary structure of the target RNA is usually unknown, we need to predict RNA secondary structure from the base sequence. Here, we use RNAshapes (24) to perform the RNA secondary structure prediction task. Starting from the base sequence, RNAshapes computes an ensemble of representative secondary structures rather than the single best-folded one. RNAshapes categorizes RNA secondary structure into five levels to abstract certain structural details, in which a higher level represents more abstraction of the structural profiles. Here, we use the first-level abstract representation (i.e. the most detailed representation) of the RNA secondary structure predicted by RNAshapes. In addition, as it has been shown in the literature that the length of 150 nucleotides has the best performance in predicting the secondary structure of mRNAs (28), we consider 150 more nucleotides from each viewpoint region in both directions (i.e. upstream and downstream). We scan the extended region using a sliding window of 150 nucleotides and with a step size of 37 nucleotides (which is approximately 25% of the window size), and then predict the secondary structures in each sliding window, in which up to three most probable secondary structures within 10% of the minimum free energy are kept. Note that a similar prediction and scanning scheme is used in (10) to encode RNA secondary structure.

With the predicted secondary structures, now each position in the RNA document can be represented by one of six elements, i.e. S, M, H, I, B and E, which stand for stem, multiloop, hairpin loop, internal loop, bulge and external regions, respectively. We then construct the RNA dictionary and the count vector (the RNA word is counted as long as the sliding window overlaps the viewpoint area for any predicted secondary structure in the ensemble) to construct the RNA secondary structural profiles using the same procedure as described in Figure 2. Since the RNA document of secondary structure has two more elements than that of base sequence, we adopt slightly longer RNA words (i.e. 8-mers) to constitute the RNA dictionary in the 2D case.

### Integrating the RNA tertiary structural profiles

RNA tertiary structure is associated with many RNA functions regulated by RBPs (34). However, predicting RNA tertiary structure from the base sequence is a challenging task in computational biology (27), which makes it difficult to study the impact of RNA tertiary structure on RBP binding behaviors. On the other hand, the currently available databases of the atomic resolution RNA tertiary structures and RNA–protein complexes can still provide a useful resource for elucidating the RNA–protein interactions. Here, we use JAR3D (25,26) to extract probable tertiary structural motifs from RNA 3D Motif Atlas (R3DMA, version 1.13, which contains 253 representative hairpin loop motifs and 276 representative internal loop motifs) (27), given the corresponding RNA base sequence and secondary structural information.

R3DMA is a comprehensive collection of the RNA tertiary structural motifs of hairpin and internal loops derived from a nonredundant set of high-resolution RNA tertiary structures. RNA tertiary structural motifs are recurrent modules that appear in the tertiary structure and are necessary components for RNA folding and various biological functions (35). More precisely, they are unstructured hairpin and internal loops (with respect to the secondary structure) with well-defined geometric arrangements of interacting nucleotides (with respect to the tertiary structure) (27). Thus, the tertiary structural motifs may involve atomic-resolution contacts that are distant in the secondary structure. Several featured examples of the RNA tertiary structural motifs are shown in Supplementary Figure S2. Motifs in R3DMA are further clustered into several groups based on the maximum cliques. Depending on R3DMA, JAR3D is a computational tool that predicts probable tertiary motifs of the hairpin and internal loops in a given RNA.

**Figure 3.**
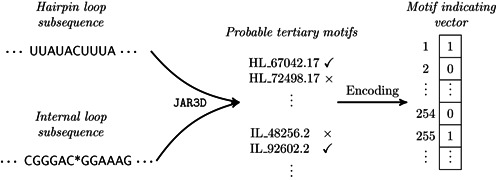
Schematic illustration of constructing the RNA tertiary structural profiles. The RNA tertiary structural motifs are predicted by JAR3D based on RNA 3D Motif Atlas (R3DMA) (25,26).

The procedure of constructing the RNA tertiary structural profiles is illustrated in Figure 3. Given a target RNA, we first predict its probable secondary structures using RNAshapes, following the same process as described in the previous section. Next, we look up all the hairpin and internal loops that overlap the viewpoint region, and then feed these selected loops into JAR3D to calculate the probabilities of folding into the corresponding tertiary structural motifs. We take zero as the cut-off score (36) to select probable tertiary structural motifs, i.e. only those with the prediction scores above zero are chosen. After that, we encode RNA tertiary structure into an indicating vector (referred to as the *motif indicating vector*) of 529 dimensions, corresponding to 253 hairpin loop motifs and 276 internal loop motifs in R3DMA v1.13. Each element in the indicating vector defines whether the nucleotide sites can fold into the corresponding tertiary structural motif.

### Predicting RBP binding sites using multimodal deep belief networks (DBNs)

In the previous sections, we have introduced the procedures of encoding RNA primary sequence, predicted secondary and tertiary structures to construct individual types of structural profiles. Next, we describe how to integrate them into a unified representation using a multimodal DBN model.

#### Multimodal DBNs

**Figure 4.**
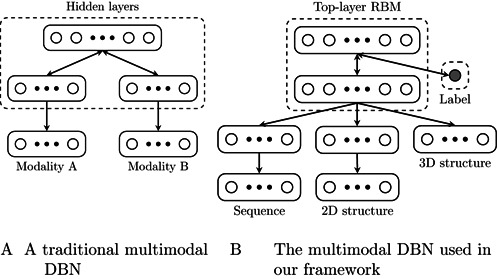
Schematic illustration of the multimodal DBN architecture. (**A**) A traditional multimodal (bimodal) DBN. (**B**) The multimodal DBN used in our framework, the overall architecture of which is (*d*_1_,*d*_2_) – (2000, 1000, 529) – 3000 – 3000, where *d*_1_ and *d*_2_ denote the dimensions of the count vectors for the primary sequence and secondary structural profiles, respectively. The label vertex attached to the top layer is used to generate the sequence and structural motifs in our test.

The multimodal DBN is a specific form of the deep learning architecture that is used to capture common latent features from the multimodal input data. For a dual-wing multimodal (also referred to as bimodal) DBN shown in Figure 4(A), its joint probability density function of the visible variables is defined by

(5) \begin{eqnarray*} p(\mathbf {v}_l,\!\mathbf {v}_r)= \sum _{{\mathbf {h}_l^{1},\mathbf {h}_r^{1},\mathbf {h}^{2}}}p(\mathbf {v}_l|\mathbf {h}_l^{1})\cdot p(\mathbf {v}_r|\mathbf {h}_r^{1})\cdot p(\mathbf {h}_l^{1},\mathbf {h}_r^{1},\mathbf {h}^{2}), \end{eqnarray*}

where the subscripts *r* and *l* mean that the variables are in the right and left wings (i.e. the bottom RBMs) of the DBN, respectively, and the superscript *i* (*i* = 1, 2) means that the variable is in the *i*th layer of the DBN, and $p(\mathbf {h}_l^{1},\mathbf {h}_r^{1},\mathbf {h}^{2})$ represents the joint probability density function of the top-layer RBM. Here, we treat the primary sequence, secondary and tertiary structures as individual modalities of the target RNA. After constructing the base sequence and secondary structural profiles using the replicated softmax, we integrate them with the predicted tertiary structural profiles using a standard DBN, which results in a multimodal DBN (see Figure 4(B)), in which the bottom two wings are the replicated softmax instead of RBMs in the traditional multimodal DBNs.

In our multimodal DBN model, we roughly halve the dimension of each input modality for the dimension reduction purpose and use 2000 and 1000 RNA topics to encode the base sequence and secondary structure, respectively. Then these 3000 latent RNA topics are concatenated with the predicted tertiary structural profiles (i.e. the 529-dimensional motif indicating vector), which constitute the hybrid layer in the network. Furthermore, we stack another two RBMs, each of which has a 3000-dimensional hidden layer. For each RBP, the overall architecture of the multimodal DBN is (*d*_1_,*d*_2_) – (2000, 1000, 529) – 3000 – 3000, where *d*_1_ and *d*_2_ stand for the dimensions of the count vectors for the primary sequence and secondary structural profiles, respectively. Note that except the dimensions *d*_1_ and *d*_2_, we use the same network architecture for all RBPs in our tests. The training and implementation details of our deep learning framework are provided in Supplementary Section S4.

### Generating RBP binding motifs from multimodal DBNs

Our multimodal DBN is a generative model in nature, which enables us to generate RBP binding motifs directly from the trained model. There are two phases to generate the binding motifs from our framework, including the training and sampling phases. In the training phase, we perform the training procedure that is similar to the pretraining process in RBP binding site prediction (see Supplementary Section S4). In particular, we follow the same principle of (14) and pretrain the multimodal DBN layer-by-layer, while training the top-layer RBM with the input layer catenated by a label vertex, which denotes whether the nucleotide sites are bound or not (see Figure 4(B)). In this case, the multimodal DBN actually models the joint distribution of the RNA base sequence and structural profiles together with its label, i.e. *P*(1*D*, 2*D*, 3*D*, *label*). In the sampling phase, we first clamp the label vertex to be one, and conduct the mean-field based Gibbs sampling in the top-layer RBM. Then the ancestral sampling method (37) is applied to downward generate the RBP binding motifs.

To generate the specific RBP binding motifs, we also need to eliminate the background motifs that appear universally in both bound and unbound scenarios. To achieve so, we also compute the expected sequence and structural profiles of the unbound RNA sites by clamping the label vertex to be zero. After that, the difference of these two probabilities, i.e. Δ*P* = *P*(1*D*, 2*D*, 3*D*|*label* = 1) − *P*(1*D*, 2*D*, 3*D*|*label* = 0), is calculated, which can be treated as the likelihood of the RBP binding motifs modeled by our framework. Based on Δ*P*, we sample from the expected RNA word distribution when clamping *label* = 1 in a rejection-sampling-liked manner, i.e. at each sampling step, the RNA word with the probability difference larger than a threshold δ is kept; otherwise, it is dropped. More implementation details of generating RBP binding motifs can be found in Supplementary Section S4.

## RESULTS

We validated the performance of our deep learning framework on 24 datasets of the HITS-CLIP-, PAR-CLIP- and iCLIP-derived RBP binding sites, in which 23 datasets were derived from doRiNA (38), and the remaining one which measured the PTB binding sites by HITS-CLIP was derived from (39). The list of RBP names is shown in Table 1, where the Ago1–4 and IGF2BP1-3 datasets contained binding sites of several RBPs, and the four datasets, i.e. ELAVL1 HITS-CLIP, ELAVL1 PAR-CLIP (A), ELAVL1 PAR-CLIP (B) and ELAVL1 PAR-CLIP (C), contained ELAVL1 binding sites derived by different experimental techniques. The preprocessed datasets were derived from (10), which included both positive samples (i.e. the RBP binding sites identified by the CLIP-based experiments) and negative samples (i.e. the unbound RNA sites created by shuffling the positions of bound sites). The descriptive statistics of these 24 RBP datasets is provided in Supplementary Table S1.

**Table 1.** AUROC performance of predicting RBP binding sites

RNA-binding protein	GraphProt	mDBN-	mDBN+
ALKBH5 PAR-CLIP	0.680	0.686	**0.714**
C17ORF85 PAR-CLIP	0.800	0.817	0.820
C22ORF28 PAR-CLIP	0.751	0.783	0.792
CAPRIN1 PAR-CLIP	0.855	0.825	0.834
Ago2 HITS-CLIP	0.765	0.805	0.809
ELAVL1 HITS-CLIP	0.955	0.964	0.966
SFRS1 HITS-CLIP	0.898	0.927	0.931
HNRNPC iCLIP	0.952	0.961	0.962
TDP43 iCLIP	0.874	0.874	0.876
TIA1 iCLIP	0.861	0.888	0.891
TIAL1 iCLIP	0.833	0.867	0.870
Ago1–4 PAR-CLIP	0.895	0.872	0.881
ELAVL1 PAR-CLIP (B)	0.935	0.956	0.961
ELAVL1 PAR-CLIP (A)	0.959	0.965	0.966
EWSR1 PAR-CLIP	0.935	0.964	0.966
FUS PAR-CLIP	0.968	0.979	0.980
ELAVL1 PAR-CLIP (C)	0.991	0.994	0.994
IGF2BP1-3 PAR-CLIP	0.889	0.872	0.879
MOV10 PAR-CLIP	0.863	0.831	**0.854**
PUM2 PAR-CLIP	0.954	0.965	0.971
QKI PAR-CLIP	0.957	0.981	0.983
TAF15 PAR-CLIP	0.970	0.980	0.983
PTB HITS-CLIP	0.937	0.879	**0.983**
ZC3H7B PAR-CLIP	0.820	0.786	0.796

*mDBN-* stands for the multimodal DBN that only integrates the RNA base sequence and secondary structural profiles, while *mDBN+* stands for the framework that integrates the RNA base sequence, secondary and tertiary structural profiles. The reported AUROC score was averaged over the 10-fold cross-validation process. The AUROC scores dropped by >2% after eliminating RNA 3D structural information are noted in bold.

### Predicting RBP binding sites

We first used the 10-fold cross-validation procedure with the standard *area under receiver operator characteristic* (AUROC) to evaluate the performance of our method on predicting RBP binding sites. We compared the prediction performance of our deep learning framework with that of the state-of-the-art method GraphProt (10). We also adopted the *relative error reduction* that has been commonly used for comparing the prediction performance of two different approaches (10), which is defined by (*c*′ − *c*)/(1 − *c*), where *c* denotes the baseline performance and *c*′ is the performance of the new method. Table 1 summarizes the prediction performance comparison between GraphProt and our framework, which indicates that our model yielded comparable or superior performance to that of GraphProt. In particular, among 24 RBPs, our method outperformed GraphProt for 19 RBPs, with an average relative error reduction of 22%, the median relative error reduction of 21% and the largest relative error reduction of 73% (achieved for the PTB dataset). Based on the prediction performance provided in (10), we further concluded that our framework outperformed RNAcontext (11), a weaker model than GraphProt, for all 24 RBPs with an average relative error reduction of 44%. Since we used the same datasets as in (10), this comparison was fair and reasonable.

### Impacts of RNA structure on RBP binding

In addition to predicting new candidate RBP binding sites, the proposed deep learning framework enables us to investigate the impacts of RNA structure on RBP binding behaviors, i.e. whether and how much the structural profiles contribute to the RNA–protein interactions. We used the following procedure to investigate this problem: After training the multimodal DBN for classification, we provided the network with partially structural information to study the influence of the missing structural profiles on the prediction performance.

**Figure 5.**
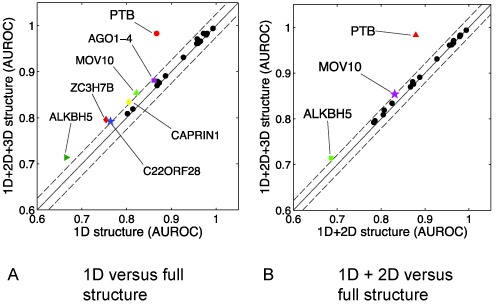
Comparison of the prediction performance between models with partially and fully structural profiles. The prediction performance was evaluated in terms of AUROC. The x-axis and y-axis represent the prediction performance of the models with partially and fully structural information, respectively. The points representing the results with similar performance are located near the solid diagonal line and the points in the area between two dashed lines have the performance difference smaller than 2%. RBPs with significant performance difference (i.e. with AUROC difference >2%) between the partially- and fully-structured models are marked with colored nodes. (**A**) Performance comparison between models with only base sequence and fully structural profiles. (**B**) Performance comparison of the model with base sequence and secondary structural profiles versus fully-structured model.

Most previous work (10–11,13) only focuses on studying the influence of the RNA secondary structural features on RNA–protein interactions. Here, we first studied the overall secondary and tertiary structural preferences of RBP binding by comparing the prediction performance between the fully structured and sequence-only models. For the sequence-only model, we removed the inputs in the secondary and tertiary structural modalities. As shown in Figure 5(A), we observed noticeable drop in performance (with AUROC score reduced by >2%) in seven RBPs, i.e. ALKBH5, AGO1–4, C22ORF28, CAPRIN1, MOV10, PTB and ZC3H7B. This result implies that RNA (secondary and tertiary) structure can play an important role in RBP binding.

We further investigated the contribution of the RNA tertiary structural features to the RBP binding preferences. We removed the tertiary structural profiles and let the deep learning model only take the base sequence and secondary structural profiles as input. After that, we compared the prediction performance with that of the fully structured model. As shown in Figure 5(B), we observed a noticeable drop in performance (with AUROC score reduced by >2%) in three RBPs: ALKBH5, MOV10 and PTB. Note that for other 21 proteins, we cannot rule out the possibility of their binding preferences attributed by the RNA tertiary structural features. The lack of difference in AUROC scores may be due to the current insufficient number of RNA tertiary structural motifs for these 21 RBPs available in R3DMA, which resulted in the limited prediction power in our model.

### Discovering potential RBP binding motifs

Discovering the potential sequence and structural binding motifs of RBPs may provide useful biological hints for studying the RBP regulation mechanisms. Here, we generated the potential sequence and structural motifs of RBP targets using the procedure described in Section ‘Materials and Methods’. Test results are summarized in Tables 2 and 3.

**Table 2.** Predicted RBP binding motifs with literature evidence

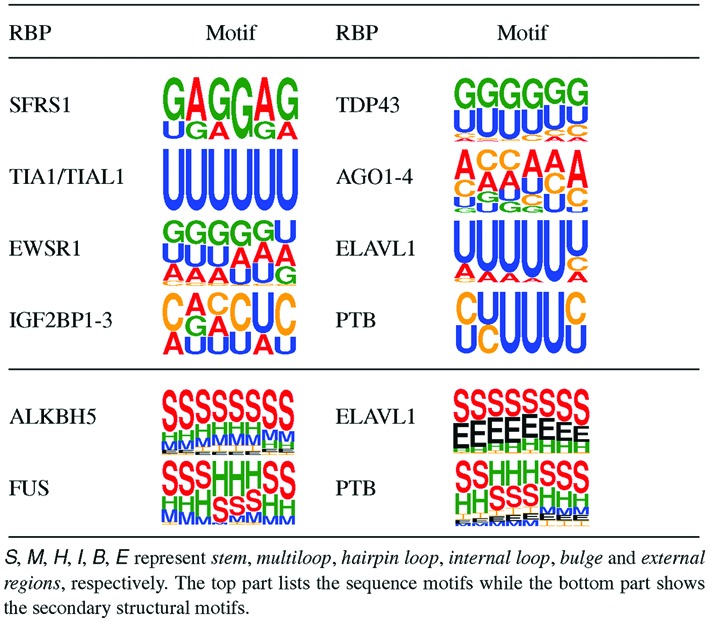			

**Table 3.** Top five potential tertiary structural binding motifs predicted by our model for ALKBH5, MOV10 and PTB

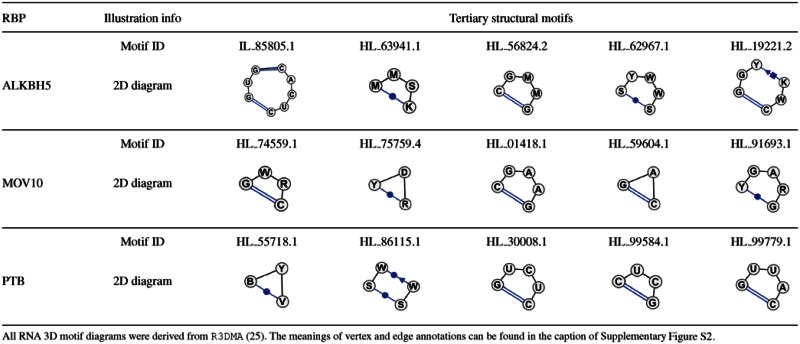						

#### Identifying the potential base sequence and secondary structural binding motifs

We first compared the predicted sequence binding motifs with those identified in the literature. Note that for fairness and rationality, here the comparing literature (for both sequence and secondary structure cases) was chosen only if it is different from that accompanying with the training samples we used. The enriched motifs of AGO1–4 identified in previous studies agreed with our results in Table 2. Particularly, AGO family proteins present a 5-mer binding motif depleted at miRNA bound sites, i.e. strong consensus base A at both ends and weak conservative bases A, C, U in the middle (40). The binding preferences of TIA1/TIAL1 and ELAVL1 captured by our framework showed a high level of motif similarity with the previously reported consensus motifs of AU-rich or U-rich elements (41,42). EWSR1 recognizes and binds to G-rich and GGU-rich elements (43,44), conforming to the motif captured by our model. SFRS1 was predicted to bind in regions with an over-represented GA-rich motif, which closely matched the consensus areas validated by the previous studies (45). IGF2BP1-3 have been identified with the common binding sites in CAU and G-less patterns (7), which displayed great agreement with the consensus motif found by our model. TDP43 binds to a consensus (UG)_*n*_ motif in both 3′ UTR and long intronic sequences, regulating pre-mRNA splicing or RNA transport and translation (46), and our model also generated the motif containing UG repeats. PTB has been described in (47) to bind to a polypyrimidine tract with the help of the RRM domains (48), which also matched the UC-rich motif identified by our model.

Various studies have validated that double-stranded RNA sites can restrict RBP binding by their stem structures, and the ubiquitous binding sites lie in single-stranded regions with relatively high affinity (49,50). These observations were consistent with our finding that a large fraction of RBPs tended to bind in unpaired regions (see Table 2). Previous experimental studies on certain RBPs have confirmed this finding. For example, ALKBH5 and ELAVL1 prefer to bind at single-stranded RNA sites during gene regulation (42,51). PTB interacts with the internal ribosomal entry site (IRES) in the 5′ or 3′ UTR, forming stem loops in both viral genome and cellular mRNAs (52), which agreed with the secondary motif identified by our model (Table 2). The FUS binding motif was found to have paired structure at the borders and hairpin loop at the center in our framework. There is also related evidence (53) indicating that FUS may prefer an AU-rich stem loop in non-neuronal cells while this result may not be so reliable in the CHIP studies in neural cells (46,53). On the other hand, no significantly specific binding motif recognition can imply that probably it also needs certain conformational features for RBP binding (46).

To check how the input RNA secondary and tertiary structural profiles contributed to the extraction of structural binding motifs in our framework, we performed an additional test on PTB by introducing a fraction of unstructured RNA sequences (i.e. bound and unbound sites devoid of the structural profiles) during the motif generating process. The test result showed that by inactivating the structural profiles for a fraction (20%) of training samples, our framework lost certain ability to fully discover the structural binding preferences of PTB, but still grasped some secondary structural specificities. More details of this test can be found in Supplementary Section S7.

#### Identifying the potential tertiary structural binding motifs

We also identified the highest scored tertiary structural binding motifs for three RBPs, i.e. ALKBH5, MOV10 and PTB, which were shown to own tertiary structural specificities for their binding targets based on the tests performed previously. Based on the probability difference Δ*P* of the RNA tertiary structural profiles for each RBP, we selected the top five potential tertiary structural binding motifs with the largest difference values. Detailed case studies on these three RBPs are provided in the next section.

We also checked the consistency between the derived tertiary structural binding motifs and the corresponding sequence and secondary structural features, taking PTB as an example. As summarized in Supplementary Table S3, the consistency was confirmed based on the following observations. First, the fraction of C and U bases in the top five motifs was high (≥60%), which was in line with the fact that PTB has CU-rich sequence binding motifs (see Table 2). Second, all these top five 3D motifs had hairpin loops, which were consistent with the PTB 2D binding motif (i.e. hairpin-loop-rich) derived by our model (see Table 2). In addition to the fact that the derived base sequence and secondary structural binding motifs of PTB have been shown to agree with the evidence in the literature, the above consistency examination provides another verification on the rationality of the potential RBP binding motifs derived by our method.

### Case studies on the impacts of RNA tertiary structure on RBP binding

Here, we present detailed discussions on the potential influence of RNA tertiary structure on RNA–protein interactions for ALKBH5, MOV10 and PTB.

ALKBH5 has been recognized as an important demethylase of N^6^-methyladenosine (m^6^A), which is referred to the methylation of the adenosine at the nitrogen-6 position (51,54–55). The RNA methylation m^6^A is the most abundant modification in mammalian mRNAs and lncRNAs, and has been found to be related to various aspects of RNA functions (55,56). The structural biology studies have validated that ALKBH5 interacts and catalyzes the m^6^A methylation of single-stranded RNAs (51,56), and it has been strongly believed that the N^6^-methylation can change RNA tertiary structure (55). In particular, the m^6^A modification can weaken RNA secondary structure and change tertiary interactions, such as base triples or Hoogsteen interactions, that involve the hydrogen bonds connected to the N^6^ proton (55,57). In addition, it has been hypothesized that m^6^A sites located in the hairpin stems can form a major groove to facilitate the RNA–protein interactions (58,59). Probably the RNA tertiary structural motifs associated with the nucleotide sites of m^6^A methylation can be used as the implicit indicators of RBP target sites. This may explain the improvement of prediction performance for ALKBH5 after incorporating the predicted RNA tertiary structural profiles (see Figure 5(B)). Note that the relatively poor prediction performance for the ALKBH5 dataset (for both GraphProt and our framework, see Table 1) may be attributed to the lack of m^6^A methylation information (which are probably related to both RNA secondary and tertiary structures) incorporated in the prediction model.

MOV10 has been known as a putative RNA helicase, which involves various RNA-mediated functions through the RNA-induced silencing complex (RISC). The recent study in (60) has indicated that MOV10 may act as an RNA clearance factor to remove proteins and resolve RNA structures from 3′ UTRs to facilitate the UPF1-regulated mRNA degradation. In (61), it has been found that, although several sequence motifs are enriched in MOV10 binding sites, these motifs are only observed with a small number of occurrences. This observation suggests that the RNA primary sequence features are not the dominant factor, and possibly the secondary and tertiary structures are also involved in the MOV10 binding preferences. Our prediction results on the binding sites of MOV10 (see Figure 5(B)) are consistent with this hypothesis.

In our prediction results of PTB, integrating the predicted RNA tertiary structural features yielded a much higher prediction accuracy than that of using only RNA primary sequence and secondary structural profiles, with the AUROC score improved from 0.879 to 0.983. As indicated by its name (i.e. polypyrimidine tract-binding), PTB prefers binding to polypyrimidine sites, and plays important roles in many functions of mRNA metabolism, such as splicing regulation, internal ribosomal entry site (IRES) mediated translation and RNA localization (52). The fact that the RNA tertiary structural profiles can significantly contribute to the RBP binding site prediction agrees well with the previous studies on the structure–function relationships of PTB and the mechanisms of its interactions with RNAs (52,62). For example, it has been shown that the interaction between PTB and the IRES of an mRNA is highly correlated with the tertiary structure of the IRES elements (52,62). In addition, the structural studies of PTB have shown that its two RRM domains bound to the CU-rich sites of a U1 snRNA form a special spatial arrangement to prevent U1 from interacting with the downstream spliceosomal elements (63). In this situation, the tertiary structure of U1 snRNA may play an important role in the binding preferences of these two RRM domains of PTB.

### A case study of the predicted PTB–RNA complex structure

PTB consists of four RRM domains (64). To further investigate how the RNA motif structure can be fitted into the protein, we tried to examine the structural details of the interactions between PTB and the top RNA 3D motifs derived from our model. Unfortunately, the experimentally-determined structures of these complexes are quite scarce. To our best knowledge, the only available structures of the RNA–protein complex involving PTB are comprised of each domain of the protein and a CUCUCU oligonuleotide which does not have an explicit tertiary structure. Thus, we used HADDOCK (65,66) to dock our derived RNA 3D motifs to the four domains of PTB respectively and examined the details of the docked complex structures.

The structures of the oligonucleotide and each domain of PTB were extracted from model 1 of the corresponding NMR complex structure. The PDB IDs of RRM1 and RRM2 domains are 2AD9 and 2ADB, respectively. The PDB ID 2ADC contains both RRM3 and RRM4 domains. The corresponding conformations of oligonucleotide CUCUCU were first docked back to the individual RRM domains to confirm if the docking platform was reliable for this test. The residues involved in the interactions between PTB and the RNA in the complex were defined as the active sites during the docking process. The docking results of RRM1, RRM2 and RRM3 generated by HADDOCK displayed RNA binding patterns that were quite similar to those determined by NMR (overall backbone RMSD values were 0.832 Å for RRM1, 0.673 Å for RRM2 and 0.723 Å for RRM3, respectively), giving us confidence to continue the docking experiments. Meanwhile, these docked complex structures for the domains RRM1, RRM2 and RRM3 were used as positive controls in the latter tests. On the contrary, the complex structure of the RRM4 domain with the docked RNA was distinct from the original NMR structure, whose binding might be influenced by its interaction with RRM3 (64). Thus, the docking results involving RRM4 were abandoned in the following tests. After that, the five derived RNA 3D motifs with the highest binding scores according to our framework (see Table 3) were tested, whose structures were extracted from the corresponding PDB files of the motifs with the lowest discrepancy according to R3DMA. The active sites were remained the same as those in the positive controls. Since a longer RNA could introduce more interactions beyond the active sites and thus result in the decrease of the total energy, here we calculated the *docking efficiency*, which is defined as –E/M, where E represents the total energy and M represents the molecular weight of all nucleotides involving the polar contacts with the protein.

The results showed that four of the top five motifs presented descent docking efficiency that were comparable to the positive controls (see Supplementary Table S4), suggesting that the score of the binding motif calculated by our framework may indicate the binding affinity to some extent. In addition, the enrichment pattern of pyrimidines was found when measuring the ratios of C and U among all the nucleotides involved in the interactions with PTB (see Supplementary Table S5), which indicated that our docked complex structures were probably reasonable. As presented in Supplementary Table S4, four of the top five motifs were successfully docked to at least one domain through the conserved binding residues without serious steric clash between atoms.

**Figure 6.**
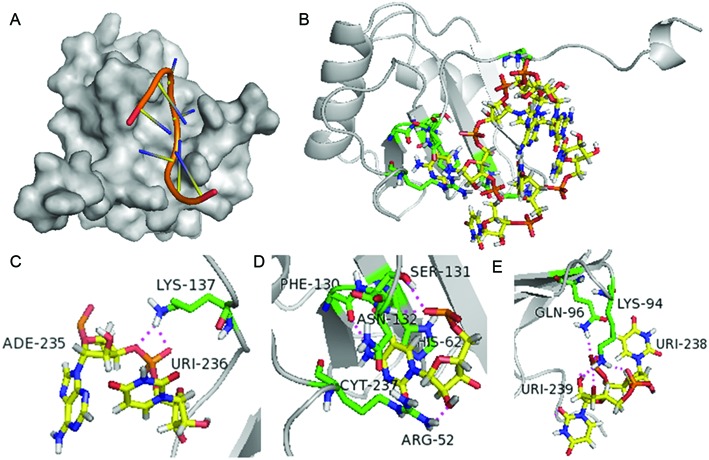
An example of the docked structure of PTB in complex with the top RNA 3D motifs derived by our model, in which the docked structure of the RRM1 domain of PTB in complex with the motif HL_86115 was predicted by HADDOCK (58,65). (**A**) The surface representation of the RRM1 domain showing how the RNA motif with a 3D structure fitted into the binding groove. (**B**) Overview of the docked structure of RRM1 domain in complex with the RNA 3D motif, in which the nucleotides and residues involved in the intermolecular interactions are shown with sticks. Close views of the binding sites of (**C**) A235 and U236, (**D**) C237, (**E**) U238 and U239 are presented in detail, respectively. The potential hydrogen bonds (predicted by PyMOL (78)) are indicated in magenta dashed lines.

The docking results may provide useful hints for understanding the intermolecular recognition between PTB and RNA. An example of the docked structure between the derived RNA 3D motif and PTB is given in Figure 6, in which the docked structure of the motif HL_86115 in complex with the RRM1 domain is shown. Certain interactions identified in the docked structure were consistent with the experimentally-determined structure (67), including the stacking force between His62 and C237 and the hydrogen bond between Phe130 and the amino group in C237. In addition, some novel interactions were observed. For instance, Lys94 or Gln96 on the second beta-sheet of RRM1 also displayed hydrogen bonds with the RNA motif and might also contribute to RNA binding, which suggests that different RNA binding patterns may also appear when PTB is bound to a well-structured RNA. As PTB plays a suppressing role in splicing along with other cofactors (64), the bound RNA may not always be a linear single strand but a structured motif bound together with other factors. Thus, it is reasonable to hypothesize that the specific RNA tertiary motifs also affect their binding affinities. Overall, these docking results might provide useful hints for understanding the post-transcriptional gene regulation of PTB.

In practice, it is generally expensive and time-consuming to experimentally study the atomic interactions between the derived RNA 3D structural motifs and PTB. Our docking procedure provided an alternative and cheap way to investigate the atomic details of their potential interactions. The motivation of our docking studies was mainly to offer a more detailed view about the RNA–PTB interactions predicted by our framework, rather than providing a direct validation of the interactions between the derived RNA 3D motifs and PTB. Thus, our docking results cannot rule out the possibility that PTB can also bind to other RNA 3D motifs that were not selected in the top list. Without experimental validation, our docking strategy can only provide a limited solution for investigating the atomic interactions between PTB and the derived RNA 3D structural motifs.

### Predicting PTB binding sites in the IRES regions

An internal ribosome entry site (IRES) segment is a nucleotide sequence that attracts the eukaryotic ribosomal translation initiation complex to facilitate the translation initiation without the usual 5′-terminal 7mG cap structure (68), and PTB has been shown to interact with the IRES regions in the translation initiation process (64). Meanwhile, it has been known that RNA tertiary structure of IRES is essential in IRES–protein interactions and the corresponding functions of IRES (69–74). Thus, we hypothesize that RNA tertiary structure can play an important role in PTB–IRES interactions. Under this hypothesis, we expected to observe better performance in predicting the PTB binding sites for the IRES regions after incorporating RNA 3D structural information. To validate this speculation, we ran our approach and GraphProt for the known IRES regions available from the database IRESite (68), and evaluated the prediction performance before and after integrating RNA 3D structural profiles. Note that though the IRES regions can interact with different RBPs, here we mainly focused on the case study of PTB to demonstrate the necessity of integrating RNA tertiary structural information into the prediction model.

**Figure 7.**
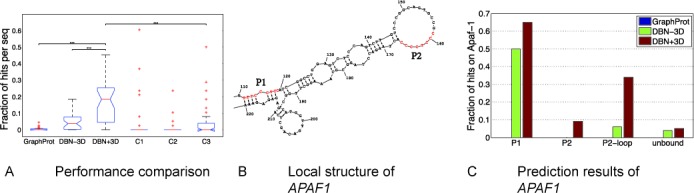
Results on predicting the PTB binding sites of the IRES regions. (**A**) Overall performance comparison (box plot), in which *DBN+3D* and *DBN-3D* represent the DBN models with and without RNA 3D structural profiles, respectively. The first three boxes represent the prediction results in the IRES regions, while the last three boxes represent the prediction results on the negative control dataset, in which *C1*, *C2* and *C3* denote the control tests for GraphProt, DBN-3D and DBN+3D, respectively. The Student's *t*-test was used to compare two different prediction methods. ‘***’ means that the *P*-value of the corresponding comparison was less than 0.001. (**B**) Local secondary structure of *APAF1*. Here we only present the local secondary structure of *APAF1* that covers the P1 and P2 sites (shown in red), which are the experimentally-verified PTB binding sites. The whole secondary structure of *APAF1* is provided in Supplementary Section S9. The figure was generated using RNAstructure (79). (**C**) Prediction results of *APAF1*. The first three labels represent the prediction results of the scanning windows overlapping the P1 and P2 sites, and the hairpin loop covering the P2 sites, respectively. The last label represents the prediction result of the remaining sites, which was considered as the unbound region. The faction of hits was defined as the ratio of the number of predicted bound windows versus the total number of scanning windows in the corresponding region.

We downloaded a set of 40 IRES segments in *Homo sapiens* from (68) that have been experimentally verified, and then predicted possible PTB binding sites in these IRES regions. More details of the tests can be found in Supplementary Section S9. Note that these IRES segments were not found in the CLIP-seq dataset of PTB that we used as training data, and among these 40 IRES regions, 13 have been validated experimentally with PTB binding. We also performed an additional negative control test on another set of 40 mRNA subsequences that did not overlap any of the 40 IRES segments (see Supplementary Section S9 for more details). Figure 7(A) shows the overall test results. Based on the tests on both IRES segments and negative control dataset, we had the following observations: (i) all three prediction approaches, including GraphProt, DBN-3D (i.e. our DBN model without RNA 3D structural information) and DBN+3D (i.e. our DBN model with RNA 3D structural information), yielded low enrichment of the predicted binding sites on the negative control dataset; (ii) GraphProt yielded similar prediction results on both IRES segments and negative control dataset; (iii) our deep learning method incorporating RNA 3D structural information showed a significantly higher enrichment of the predicted binding sites in the IRES regions than the negative control dataset (with *P*-value = 5.57 × 10^−6^); (iv) our DBN model integrating RNA 3D structural information achieved a much higher fraction of hits on the IRES segments than GraphProt and DBN-3D (with *P*-values 3.59 × 10^−12^ and 1.53 × 10^−7^, respectively). Based on these observations, we concluded that: (i) the increased number of hits in the IRES regions produced by DBN+3D was not attributed to the false-positive issue, in other words, our deep learning method did not suffer from the false positive problem; (ii) compared to GraphProt and DBN-3D, which did not consider RNA 3D structural information, DBN+3D yielded a much higher enrichment of the predicted binding sites on the IRES segments, which was probably due to the PTB recognition preference in RNA 3D structure in these IRES regions. All these results further demonstrate the necessity of integrating RNA 3D structural information into the model of predicting RBP binding sites.

We also examined individual prediction results of the 13 IRES segments, in which the PTB binding has been validated experimentally in the literature (see Supplementary Figure S5(A)), and provided the predicted PTB binding sites for the other 27 IRES regions (see Supplementary Figure S5(B)). More details about these individual results can be found in Supplementary Section S9. To further investigate the prediction performance of our method in more detail, we also investigated the details of the predicted binding sites in IRES segments *APAF1* and *UNR*, in which the exact PTB binding sites have been verified experimentally (70,75–76). Here we mainly used the results of *APAF1* as a case study. The prediction results of *UNR* (upstream of N-ras) can be found in Supplementary Section S9.

*APAF1* is a well-studied IRES segment that is bound to PTB, and multiple functional assays on its mutations have been performed to identify the binding patterns (70). Importantly, these studies indicate that PTB can only bind to the IRES region of *APAF1* when it folds into a correct tertiary conformation, which is achieved by *UNR* co-binding that opens the loops near the PTB binding sites (70). The experimentally-verified PTB binding sites of *APAF1* contain both P1 and P2 sites, as shown in red in Figure 7(B), in which the P1 sites are located around an internal loop while the P2 sites are located in a hairpin loop. The P2-loop sites were defined as the hairpin loop that contains the P2 sites. We examined the prediction results of four different types of regions, including the P1, P2, P2-loop regions, which contained the RNA subsequences in the scanning windows that overlapped the P1, P2 and P2-loop sites, respectively, and the unbound region, which contained the remaining sites that are not found to bind to PTB experimentally. As shown in Figure 7(C), GraphProt failed to detect any bound site in the P1 and P2 regions of *APAF1*. In contrast, our deep learning method can detect the experimentally-verified PTB binding sites in both P1 and P2 regions. In particular, for the P1 region, both DBNs with and without RNA 3D structural information can detect bound subsequences that overlapped the experimentally-verified sites. This implies that the PTB–P1 interaction may strongly prefer certain secondary structural features. In fact, the P1 sites are located in an almost double-stranded region (with only a one-nucleotide internal loop, see Figure 7(B)). Although GraphProt also considered RNA secondary structural information for binding site prediction, it was unable to discover any target site in the P1 region. Furthermore, only the DBN model with RNA 3D structural information was able to detect the PTB–P2 interaction, which indicates that probably there is certain tertiary structural preference for PTB binding in this region. For the P2 region, the number of the detected bound sites consistent with experimental validation was relatively small compared to that in the P1 region. We speculated that, due to the complicated mechanism of PTB-(*APAF1*) interactions, there may be some bias in our prediction. To demonstrate this point, we relaxed the ‘ground-truth’ bound sites to the entire hairpin loop covering the P2 sites and found that the consideration of such bias can yield a much better prediction result, as shown in Figure 7(C). We also observed that the false-positive ratio, i.e. the fraction of hits in the unbound region, was much smaller than that of the bound regions. This result further indicates that it is unlikely that our method suffered from the false-positive issue.

The test results of *APAF1* and *UNR* (see Supplementary Figure S7) provide excellent examples to explain PTB's role as a general RNA chaperone to mediate the translation of mRNAs. According to the previous experimental studies, the binding of PTB and other cofactors to these RNAs can generate a conformational change, usually the disturbance of the stem regions to release the structured regions into available loops, and make the RNA fold into a specific tertiary structure required for translation (70,72,75,77). Thus, it is reasonable to speculate that RNA tertiary structure can contribute to RBP binding preferences, which may explain the improvement of the prediction performance of our framework after integrating the RNA 3D structural profiles.

## CONCLUSION

We have developed a deep learning framework to model the binding preferences of RNA-binding proteins by integrating the primary sequence, predicted secondary and tertiary structural profiles of the target sites. Our framework considered RNA tertiary structure for RBP binding site prediction, and provided strong evidence to support the view that the RNA tertiary structural features can contribute to RBP target recognition. Tests on real CLIP-seq datasets showed that our framework can achieve the comparable or superior performance to the state-of-the-art method for predicting RBP binding sites. In addition, the structural motifs generated by our framework agreed well with the previous experimental studies in the literature, and may provide useful hints for further elucidating the molecular recognition mechanisms of RBPs.

## SUPPLEMENTARY DATA

Supplementary Data are available at NAR online.

SUPPLEMENTARY DATA

## References

[1] Keene J.D. (2007). RNA regulons: coordination of post-transcriptional events. Nat. Rev. Genet..

[2] Li X., Kazan H., Lipshitz H.D., Morris Q.D. (2014). Finding the target sites of RNA-binding proteins. WIREs RNA.

[3] Musunuru K. (2003). Cell-specific RNA-binding proteins in human disease. Trends Cardiovasc. Med..

[4] Lukong K., Chang K., Khandjian E., Richard S. (2008). RNA-binding proteins in human genetic disease. Trends Genet..

[5] Licatalosi D.D., Mele A., Fak J.J., Ule J., Kayikci M., Chi S.W., Clark T.A., Schweitzer A.C., Blume J.E., Wang X. (2008). HITS-CLIP yields genome-wide insights into brain alternative RNA processing. Nature.

[6] König J., Zarnack K., Rot G., Curk T., Kayikci M., Zupan B., Turner D.J., Luscombe N.M., Ule J. (2010). iCLIP reveals the function of hnRNP particles in splicing at individual nucleotide resolution. Nat. Struct. Mol. Biol..

[7] Hafner M., Landthaler M., Burger L., Khorshid M., Hausser J., Berninger P., Rothballer A., Ascano M., Jungkamp A.-C., Munschauer M., Ulrich A. (2010). Transcriptome-wide identification of RNA-binding protein and microRNA target sites by PAR-CLIP. Cell.

[8] Yang Y.-C., Di C., Hu B, Zhou M. (2015). CLIPdb: a CLIP-seq database for protein-RNA interactions. BMC Genomics.

[9] Pudimat R., Schukat-Talamazzini E.-G., Backofen R. (2005). A multiple-feature framework for modelling and predicting transcription factor binding sites. Bioinformatics.

[10] Maticzka D., Lange S.J., Costa F., Backofen R. (2014). GraphProt: modeling binding preferences of RNA-binding proteins. Genome Biol..

[11] Kazan H., Ray D., Chan E.T., Hughes T.R., Morris Q. (2010). RNAcontext: a new method for learning the sequence and structure binding preferences of RNA-binding proteins. PLoS Comput. Biol..

[12] Hiller M., Pudimat R., Busch A., Backofen R. (2006). Using RNA secondary structures to guide sequence motif finding towards single-stranded regions. Nucleic Acids Res..

[13] Fukunaga T., Ozaki H., Terai G., Asai K., Iwasaki W., Kiryu H. (2014). CapR: revealing structural specificities of RNA-binding protein target recognition using CLIP-seq data. Genome Biol..

[14] Hinton G.E., Osindero S., Teh Y.-W. (2006). A fast learning algorithm for deep belief nets. Neural Comput..

[15] Hinton G., Salakhutdinov R. (2006). Reducing the dimensionality of data with neural networks. Science.

[16] Hinton G., Deng L., Yu D., Dahl G., Mohamed A., Jaitly N., Senior A., Vanhoucke V., Nguyen P., Sainath T., Kingsbury B. (2012). Deep neural networks for acoustic modeling in speech recognition: the shared views of four research groups. Signal Processing Magazine, IEEE.

[17] Collobert R., Weston J., Bottou L., Karlen M., Kavukcuoglu K., Kuksa P. (2011). Natural language processing (almost) from scratch. J. Machine Learning Res..

[18] Lena P.D., Nagata K., Baldi P.F. (2012). Deep spatio-temporal architectures and learning for protein structure prediction. Advances in Neural Information Processing Systems 25.

[19] Lusci A., Pollastri G., Baldi P. (2013). Deep architectures and deep learning in chemoinformatics: the prediction of aqueous solubility for drug-like molecules. J. Chem. Inf. Model..

[20] Leung M. K.K., Xiong H.Y., Lee L.J., Frey B.J. (2014). Deep learning of the tissue-regulated splicing code. Bioinformatics.

[21] Ngiam J., Khosla A., Kim M., Nam J., Lee H., Ng A.Y. (2011). Multimodal deep learning. International Conference on Machine Learning.

[22] Srivastava N., Salakhutdinov R. (2012). Multimodal learning with deep Boltzmann machines. Advances in Neural Information Processing Systems 25.

[23] Liang M., Li Z., Chen T., Zeng J. (2015). Integrative Data Analysis of Multi-Platform Cancer Data with a Multimodal Deep Learning Approach. IEEE/ACM Trans. Comput. Biology Bioinform..

[24] Steffen P., Voss B., Rehmsmeier M., Reeder J., Giegerich R. (2006). RNAshapes: an integrated RNA analysis package based on abstract shapes. Bioinformatics.

[25] Sarver M., Zirbel C.L., Stombaugh J., Mokdad A., Leontis N.B. (2008). FR3D: finding local and composite recurrent structural motifs in RNA 3D structures. J. Math. Biol..

[26] Rahrig R.R., Leontis N.B., Zirbel C.L. (2010). R3D Align: global pairwise alignment of RNA 3D structures using local superpositions. Bioinformatics.

[27] Petrov A.I., Zirbel C.L., Leontis N.B. (2013). Automated classification of RNA 3D motifs and the RNA 3D Motif Atlas. RNA.

[28] Lange S.J., Maticzka D., Möhl M., Gagnon J.N., Brown C.M., Backofen R. (2012). Global or local? Predicting secondary structure and accessibility in mRNAs. Nucleic Acids Res..

[29] Tenenbaum J.B., Kemp C., Griffiths T.L., Goodman N.D. (2011). How to grow a mind: statistics, structure, and abstraction. Science.

[30] Fischer A., Igel C. (2012). An introduction to restricted Boltzmann machines. Progress in Pattern Recognition, Image Analysis, Computer Vision, and Applications Vol. 7441 of Lecture Notes in Computer Science.

[31] Bengio Y. (2009). Learning deep architectures for AI. Foundations Trends Machine Learning.

[32] Hinton G.E. (2002). Training products of experts by minimizing contrastive divergence. Neural Comput..

[33] Hinton G.E., Salakhutdinov R. (2009). Replicated softmax: an undirected topic model. Advances in Neural Information Processing Systems 22.

[34] Hajdin C.E., Ding F., Dokholyan N.V., Weeks K.M. (2010). On the significance of an RNA tertiary structure prediction. RNA.

[35] Nasalean L., Stombaugh J., Zirbel C.L., Leontis N.B., Walter N, Woodson S, Batey R (2009). RNA 3D structural motifs: definition, identification, annotation, and database searching. Non-Protein Coding RNAs, Vol. 13 of Springer Series in Biophysics.

[36] Zirbel C.L. (2014).

[37] Bishop C.M. (2006). Pattern Recognition and Machine Learning.

[38] Anders G., Mackowiak S.D., Jens M., Maaskola J., Kuntzagk A., Rajewsky N., Landthaler M., Dieterich C. (2012). doRiNA: a database of RNA interactions in post-transcriptional regulation. Nucleic Acids Res..

[39] Xue Y., Zhou Y., Wu T., Zhu T., Ji X., Kwon Y.-S., Zhang C., Yeo G., Black D.L., Sun H. (2009). Genome-wide analysis of PTB-RNA interactions reveals a strategy used by the general splicing repressor to modulate exon inclusion or skipping. Mol. Cell.

[40] Li J., Kim T., Nutiu R., Ray D., Hughes T.R., Zhang Z. (2014). Identifying mRNA sequence elements for target recognition by human Argonaute proteins. Genome Res..

[41] Dember L.M., Kim N.D., Liu K.-Q., Anderson P. (1996). Individual RNA recognition motifs of TIA-1 and TIAR have different RNA binding specificities. J. Biological Chem..

[42] Meisner N.-C., Hackermüller J., Uhl V., Aszódi A., Jaritz M., Auer M. (2004). mRNA openers and closers: modulating AU-rich element-controlled mRNA stability by a molecular switch in mRNA secondary structure. ChemBioChem.

[43] Nguyen C.D., Mansfield R.E., Leung W., Vaz P.M., Loughlin F.E., Grant R.P., Mackay J.P. (2011). Characterization of a family of RanBP2-type zinc fingers that can recognize single-stranded RNA. J. Mol. Biol..

[44] Takahama K., Kino K., Arai S., Kurokawa R., Oyoshi T. (2011). Identification of Ewing's sarcoma protein as a G-quadruplex DNA- and RNA-binding protein. FEBS J..

[45] Sanford J.R., Wang X., Mort M., VanDuyn N., Cooper D.N., Mooney S.D., Edenberg H.J., Liu Y. (2009). Splicing factor SFRS1 recognizes a functionally diverse landscape of RNA transcripts. Genome Res..

[46] Colombrita C., Onesto E., Megiorni F., Pizzuti A., Baralle F.E., Buratti E., Silani V., Ratti A. (2012). TDP-43 and FUS RNA-binding proteins bind distinct sets of cytoplasmic messenger RNAs and differently regulate their post-transcriptional fate in motoneuron-like cells. J. Biol. Chem..

[47] Tuerk C., Gold L. (1990). Systematic evolution of ligands by exponential enrichment: RNA ligands to bacteriophage T4 DNA polymerase. Science.

[48] Reid D.C., Chang B.L., Gunderson S.I., Alpert L., Thompson W.A., Fairbrother W.G. (2009). Next-generation SELEX identifies sequence and structural determinants of splicing factor binding in human pre-mRNA sequence. RNA.

[49] Freeberg M.A., Han T., Moresco J.J., Kong A., Yang Y.-C., Lu Z.J., Yates J.R., Kim J.K. (2013). Pervasive and dynamic protein binding sites of the mRNA transcriptome in Saccharomyces cerevisiae. Genome Biol..

[50] Li X., Quon G., Lipshitz H.D., Morris Q. (2010). Predicting in vivo binding sites of RNA-binding proteins using mRNA secondary structure. RNA.

[51] Zheng G., Dahl J., Niu Y., Fedorcsak P., Huang C.-M., Li C., Våbø C., Shi Y., Wang W.-L., and Song S.-H. (2013). ALKBH5 is a mammalian RNA demethylase that impacts RNA metabolism and mouse fertility. Mol. Cell.

[52] Auweter S., Allain F. (2008). Structure-function relationships of the polypyrimidine tract binding protein. Cell. Mol. Life Sci..

[53] Hoell J.I., Larsson E., Runge S., Nusbaum J.D., Duggimpudi S., Farazi T.A., Hafner M., Borkhardt A., Sander C., Tuschl T. (2011). RNA targets of wild-type and mutant FET family proteins. Nat. Struct. Mol. Biol..

[54] Xu C., Liu K., Tempel W., Demetriades M., Aik W., Schofield C., Min J. (2014). Structures of human ALKBH5 demethylase reveal a unique binding mode for specific single-stranded N6-methyladenosine RNA demethylation. J. Biol. Chem..

[55] Pan T. (2013). N6-methyl-adenosine modification in messenger and long non-coding RNA. Trends Biochem. Sci..

[56] Jia G., Fu Y., He C. (2013). Reversible RNA adenosine methylation in biological regulation. Trends Genet..

[57] Kierzek E., Kierzek R. (2003). The thermodynamic stability of RNA duplexes and hairpins containing N6-alkyladenosines and 2-methylthio-N6-alkyladenosines. Nucleic Acids Res..

[58] Dominissini D., Moshitch-Moshkovitz S., Schwartz S., Salmon-Divon M., Ungar L., Osenberg S., Cesarkas K., Jacob-Hirsch J., Amariglio N., Kupiec M. (2012). Topology of the human and mouse m^6^A RNA methylomes revealed by m^6^A-seq. Nature.

[59] Chandola U., Das R., Panda B. (2015). Role of the N6-methyladenosine RNA mark in gene regulation and its implications on development and disease. Brief. Funct. Genom..

[60] Gregersen L.H., Schueler M., Munschauer M., Mastrobuoni G., Chen W., Kempa S., Dieterich C., Landthaler M. (2014). MOV10 is a 5’ to 3’ RNA helicase contributing to UPF1 mRNA target degradation by translocation along 3’ UTRs. Mol. Cell.

[61] Sievers C., Schlumpf T., Sawarkar R., Comoglio F., Paro R. (2012). Mixture models and wavelet transforms reveal high confidence RNA-protein interaction sites in MOV10 PAR-CLIP data. Nucleic Acids Res..

[62] Song Y., Tzima E., Ochs K., Bassili G., Trusheim H., Linder M., Preissner K.T., Niepmann M. (2005). Evidence for an RNA chaperone function of polypyrimidine tract-binding protein in picornavirus translation. RNA.

[63] Sharma S., Maris C., Allain F. H.-T., Black D.L. (2011). U1 snRNA directly interacts with polypyrimidine tract-binding protein during splicing repression. Mol. Cell.

[64] Romanelli M.G., Diani E., Lievens P. M.-J. (2013). New insights into functional roles of the polypyrimidine tract-binding protein. Internat. J. Mol. Sci..

[65] de Vries S.J., van Dijk A. D.J., Krzeminski M., van Dijk M., Thureau A., Hsu V., Wassenaar T., Bonvin A. M. J.J. (2007). HADDOCK versus HADDOCK: new features and performance of HADDOCK2.0 on the CAPRI targets. Proteins: Struct. Funct. Bioinform..

[66] Dominguez C., Boelens R., Bonvin A. M. J.J. (2003). HADDOCK: a protein-protein docking approach based on biochemical or biophysical information. J. Am. Chem. Soc..

[67] Oberstrass F.C., Auweter S.D., Erat M., Hargous Y., Henning A., Wenter P., Reymond L., Amir-Ahmady B., Pitsch S., Black D.L. et al. (2005). Structure of PTB bound to RNA: specific binding and implications for splicing regulation. Science.

[68] Mokrejš M., Mašek T., Vopálenský V., Hlubuček P., Delbos P., Pospíšek M. (2010). IRESite — a tool for the examination of viral and cellular internal ribosome entry sites. Nucleic Acids Res..

[69] Khan D., Sharathchandra A., Ponnuswamy A., Grover R., Das S. (2013). Effect of a natural mutation in the 5’ untranslated region on the translational control of p53 mRNA. Oncogene.

[70] Mitchell S.A., Spriggs K.A., Coldwell M.J., Jackson R.J., Willis A.E. (2003). The Apaf-1 internal ribosome entry segment attains the correct structural conformation for function via interactions with PTB and unr. Mol. Cell.

[71] Sharathchandra A., Lal R., Khan D., Das S. (2012). Annexin A2 and PSF proteins interact with p53 IRES and regulate translation of p53 mRNA. RNA Biol..

[72] Grover R., Sharathchandra A., Ponnuswamy A., Khan D., Das S. (2011). Effect of mutations on the p53 IRES RNA structure: implications for de-regulation of the synthesis of p53 isoforms. RNA Biol..

[73] Cammas A., Dubrac A., Morel B., Lamaa A., Touriol C., Teulade-Fichou M.-P., Prats H., Millevoi S. (2015). Stabilization of the G-quadruplex at the VEGF IRES represses cap-independent translation. RNA Biol..

[74] Veo B.L., Krushel L.A. (2012). Secondary RNA structure and nucleotide specificity contribute to internal initiation mediated by the human tau 5’ leader. RNA Biol..

[75] Mitchell S.A., Brown E.C., Coldwell M.J., Jackson R.J., Willis A.E. (2001). Protein factor requirements of the Apaf-1 internal ribosome entry segment: roles of polypyrimidine tract binding protein and upstream of N-ras. Mol. Cell. Biol..

[76] Cornelis S., Tinton S.A., Schepens B., Bruynooghe Y., Beyaert R. (2005). UNR translation can be driven by an IRES element that is negatively regulated by polypyrimidine tract binding protein. Nucleic Acids Res..

[77] Pickering B.M., Mitchell S.A., Spriggs K.A., Stoneley M., Willis A.E. (2004). Bag-1 internal ribosome entry segment activity is promoted by structural changes mediated by poly(rC) binding protein 1 and recruitment of polypyrimidine tract binding protein 1. Mol. Cell. Biol..

[78] Schrödinger, LLC (2010). The PyMOL Molecular Graphics System.

[79] Reuter J.S., Mathews D.H. (2010). RNAstructure: software for RNA secondary structure prediction and analysis. BMC Bioinform..

