# Supplementary material for: A deep learning framework for modeling structural features of RNA-binding protein targets
Source: Nucleic Acids Res. 2015 Oct 13;44(4):e32. doi: 10.1093/nar/gkv1025 (PMC4770198; doi:10.1093/nar/gkv1025)
Supplement: SUPPLEMENTARY DATA [file supp_44_4_e32__index.html]

A deep learning framework for modeling structural features of RNA-binding protein targets — A deep learning framework for modeling structural features of RNA-binding protein targets — SUPPLEMENTARY DATA 

# A deep learning framework for modeling structural features of RNA-binding protein targets

## SUPPLEMENTARY DATA

- SUPPLEMENTARY DATA
