# Supplementary material for: A deep learning framework for modeling structural features of RNA-binding protein targets
Source: Nucleic Acids Res. 2015 Oct 13;44(4):e32. doi: 10.1093/nar/gkv1025 (PMC4770198; doi:10.1093/nar/gkv1025)
Supplement: SUPPLEMENTARY DATA [file supp_gkv1025_nar-01068-met-g-2015-File047.pdf]

Supplementary Material

A deep learning framework for modeling structural features of RNA-binding protein targets

Sai Zhang[1], Jingtian Zhou[2,*], Hailin Hu[2,*], Haipeng Gong[3,4], Ligong Chen[2], Chao Cheng[5,†], and Jianyang Zeng[1,4,†]

[1]Institute for Interdisciplinary Information Sciences, Tsinghua University, Beijing, 100084, China
[2]Department of Pharmacology and Pharmaceutical Sciences, School of Medicine, Tsinghua University, Beijing, 100084, China
[3]School of Life Sciences, Tsinghua University, Beijing, 100084, China
[4]MOE Key Laboratory of Bioinformatics, Tsinghua University, Beijing, 100084, China
[5]Department of Genetics, Institute for Quantitative Biomedical Sciences, Norris Cotton Cancer Center, Geisel School of Medicine at Dartmouth, Hanover, NH 03755, USA

S1 Supplementary elaboration on the RBM and replicated softmax models

Two examples of the RBM and replicated softmax models are shown in Fig. S1. Next, we provide more details of the replicated softmax model, which is used to encode both RNA base sequence and secondary structural profiles in our deep learning framework.

Replicated softmax is a specific graphical model to characterize the word distribution in documents based on the hidden topics [1]. In particular, we first assume that the word distribution of the document is controlled by the combination of various topics. For example, in the book *Mathematical Thought from Ancient to Modern Times* [2] with topics such as history, mathematics and philosophy, the occurrence frequencies of certain words like “Greece”, “geometry” and “Platonism” can be higher than other words like “football” and “apple”. Given a set of documents, we always want to classify them or infer their probable topics for further analysis. The replicated softmax model is designed to perform such tasks, and certain modifications can be made to use this model to characterize the RNA word distribution in RNA sequences, as described in the main paper.

*These authors contributed equally to this work.
†To whom correspondence should be addressed. Emails: chao.cheng@dartmouth.edu, zengjy321@tsinghua.edu.cn

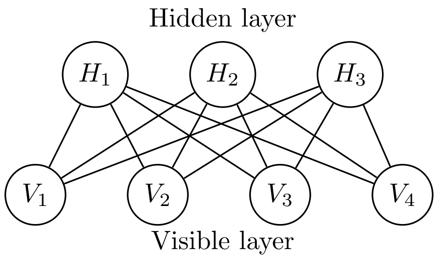

(a) RBM

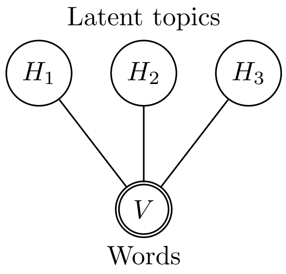

(b) Replicated softmax

Figure S1: Examples of the RBM and replicated softmax models. (a) An RBM model. (b) A replicated softmax model, in which "words" represent the input RNA base sequence or secondary structural profiles, while "latent topics" are hidden variables that represent unknown factors controlling the RNA sequence or secondary structural features.

To exemplify our notation defined in the main paper, let us consider an RNA sequence/document $\mathscr{D} = \mathsf{AAUCG}$ and the corresponding RNA dictionary $\mathscr{K} = (\mathsf{A}, \mathsf{U}, \mathsf{C}, \mathsf{G})$. Then the corresponding matrix representation for this RNA document is

$$\mathbf{V} = \begin{pmatrix} 1 & 1 & 0 & 0 & 0 \\ 0 & 0 & 1 & 0 & 0 \\ 0 & 0 & 0 & 1 & 0 \\ 0 & 0 & 0 & 0 & 1 \end{pmatrix}.$$

The energy function and the corresponding joint probability density function are defined in the main paper (see Eqs. 3 and 4, respectively). Based on these definitions, the conditional distributions of visible and hidden variables are given by

$$P(V^k = 1|\mathbf{h}) = \frac{\exp\left(\sum_{j=1}^{N} w_j^k h_j + b^k\right)}{\sum_{q=1}^{K} \exp\left(\sum_{j=1}^{N} w_j^q h_j + b^q\right)}, \tag{S1}$$

$$P(H_j = 1|\mathbf{V}) = \mathrm{sig}\left(\sum_{k=1}^{K} w_j^k \hat{v}^k + Dc_j\right), \tag{S2}$$

where $\mathrm{sig}(x)$ stands for the sigmoid function. Note that Eq. S1, which computes the probability of each RNA word appearing in the RNA document given a particular combination of latent topics (denoted as the hidden variables), is the so-called *softmax function.* Since we omit the occurrence order of the RNA words and only consider their frequencies, the *bag of words* (but not the *sequence of words*) can be generated by (replicated) sampling from the model. These two points interpret the model's name — *replicated softmax.*

S2 Featured examples of the RNA tertiary structural motifs

Figure S2 shows four examples of RNA tertiary structural motifs derived from `R3DMA` [3].

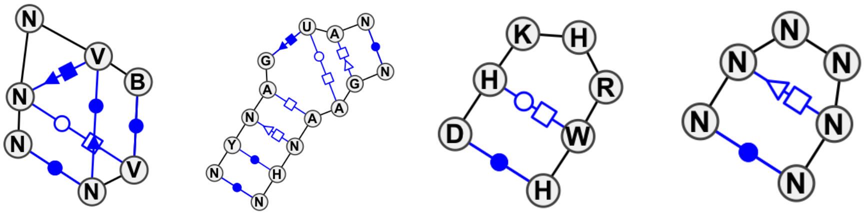

(a) C-loop (b) Sarcin-ricin (c) T-loop (d) GNRA

Figure S2: Several featured examples of the RNA tertiary structural motifs derived from `R3DMA` [3]. (a) C-loop (ID: IL_73276.9). (b) Sarcin-ricin parent motif with 15 nts (ID: IL_85647.7). (c) T-loops with two bulged bases (ID: HL_72498.17). (d) GNRA Hairpin motifs (ID: HL_67042.17). The vertex annotations *Y*, *R*, *W*, *S*, *K*, *M*, *D*, *V*, *H*, *B*, *A*, *G*, *C*, *U* and *N* stand for *pyrimidines* (`C` or `U`), *purines* (`G` or `A`), *weak* (`A` or `U`), *strong* (`G` or `C`), *keto* (`G` or `U`), *amino* (`A` or `C`), *not C*, *not U*, *not G*, *not A*, *only A*, *only G*, *only C*, *only U* and *any nucleotide*, respectively. The geometric edge annotations represent different RNA basepairs [4, 5].

S3 Descriptive statistics on the `CLIP-seq` datasets of 24 RBPs

Table S1 provides descriptive statistics on the `CLIP-seq` datasets of 24 RBPs used in our tests, including the dimensions of individual sequence and structural profiles, the number of (positive and negative) samples and the size of the validation set.

S4 Supplementary details of cross-validation, model training and implementation

In this section, we provide the details on the 10-fold cross-validation, model training and implementation of our deep learning framework.

To evaluate the performance of our model, we performed a 10-fold cross-validation procedure for all 24 RBP datasets. In particular, when performing cross-validation, we further randomly splitted the nine non-testing subsamples into two parts, including eight subsamples for pure model training and one subsample for validation. We also verified that there were rarely nearly-identical sequences in any two validation sets, since the percentage of the pairwise homologous RNA sequences in each of the 24 RBP datasets was small ($< 1\%$, computed by BLAST [6] using E-value of 0.001, see also Table S1 for details).

To train our deep learning framework, we first performed the layer-wise unsupervised learning strategy to *pretrain* the network (DBN) [7], during which the modality-specific information was eliminated, the sequence and structural profiles in different dimensions were integrated and the unified implicit representation that characterizes the RBP binding preference was constructed. To predict RBP binding sites, we further transformed the multimodal DBN to a standard neural network with the same architecture, in which

Table S1: Descriptive statistics of the `CLIP-seq` datasets. "# of +" denotes the number of positive samples, while "# of -" denotes the number of negative samples. The column "# of val" reports the average size of the validation set used in the 10-fold cross-validation procedure. The percentages of the pairwise homologous RNA sequences of these 24 RBP datasets were all less than 1% (computed by `BLAST` [6] using E-value of 0.001).

RNA-binding protein	1D	2D	3D	# of +	# of -	# of val
ALKBH5 PAR-CLIP	4,065	1,849	529	1,213	1,197	241
C17ORF85 PAR-CLIP	4,081	1,889	529	1,860	1,849	371
C22ORF28 PAR-CLIP	4,096	2,024	529	9,369	9,136	1,851
CAPRIN1 PAR-CLIP	4,096	2,002	529	8,140	7,901	1,604
Ago2 HITS-CLIP	4,096	2,073	529	48,095	44,251	9,235
ELAVL1 HITS-CLIP	4,095	1,985	529	8,595	8,436	1,703
SFRS1 HITS-CLIP	4,096	2,073	529	19,438	17,195	3,663
HNRNPC iCLIP	4,096	2,043	529	21,472	19,794	4,127
TDP43 iCLIP	4,096	2,095	529	92,031	75,079	16,711
TIA1 iCLIP	4,096	2,042	529	18,049	16,135	3,418
TIAL1 iCLIP	4,096	2,075	529	42,332	36,652	7,898
Ago1-4 PAR-CLIP	4,096	2,064	529	36,902	31,310	6,821
ELAVL1 PAR-CLIP (B)	4,096	2,036	529	27,275	23,974	5,125
ELAVL1 PAR-CLIP (A)	4,096	1,994	529	9,464	9,283	1,875
EWSR1 PAR-CLIP	4,096	1,986	529	16,292	14,720	3,101
FUS PAR-CLIP	4,096	2,029	529	34,581	31,480	6,606
ELAVL1 PAR-CLIP (C)	4,096	2,065	529	125,202	113,686	23,889
IGF2BP1-3 PAR-CLIP	4,096	2,030	529	8,539	6,838	1,538
MOV10 PAR-CLIP	4,096	2,038	529	13,793	12,987	2,678
PUM2 PAR-CLIP	4,096	1,956	529	9,116	8,227	1,734
QKI PAR-CLIP	4,096	1,943	529	10,276	9,142	1,942
TAF15 PAR-CLIP	4,094	1,890	529	7,298	6,606	1,390
PTB HITS-CLIP	4,096	2,052	529	44,574	43,700	8,827
ZC3H7B PAR-CLIP	4,096	2,090	529	20,962	20,018	4,098

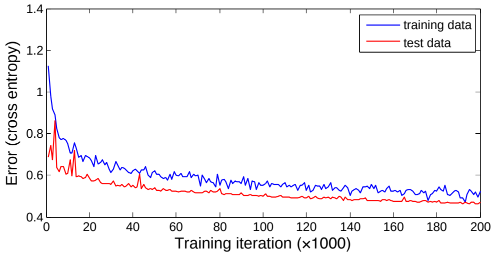

Figure S3: The error curves of the training and test data of PTB. The cross entropy was used to define the error score [8].

the parameters were initialized via the aforementioned pretraining process, and then the error back-propagation strategy was conducted to fine-tune the model parameters. Note that to keep the bottom replicated softmax models interpretable, i.e., to make the hidden layers attached to the input base sequence and secondary structural profiles always represent the corresponding RNA topics, we did not change their parameters in the back-propagation process.

We implemented our deep learning framework based on `deepnet`[1], a GPU-based python library that can be used to implement various deep learning models, e.g., deep belief networks (DBNs), deep Boltzmann machines (DBMs) and stacked autoencoders. The hyperparameters of the proposed deep learning model, including the learning rate, batch size and momentum, were set to be the default values in `deepnet` (which are also commonly used in the deep learning community), and were fixed for all RBPs. To train the RBM and replicated softmax models, we used the mean-field version of the contrastive divergence (CD) algorithm [9] and set the mini-batch size to be 10 and the learning rate to be 0.01. All the model parameters were initialized with zero-mean Gaussian distribution of standard derivation 0.01. We used the momentum method to accelerate the learning process [10], whose initial and final values were set to be 0.5 and 0.9, respectively. For a better regularization, the *weight decay* technique was conducted in the pretraining procedure by adding an extra penalty term to the log-likelihood function [10]. In particular, the L_2 penalty term was used and its coefficient was set to be 10^{-4}. After the layer-wise pretraining, we stacked another logistic layer on the top of the DBN for RBP binding site prediction. This top layer was initialized with zero-mean Gaussian distribution of standard derivation 0.01. In the error back-propagation training process, we fine-tuned the whole network except the bottom two replicated softmax layers with learning rate 0.05. In particular, in each fold of the cross-validation process, we first ran the error back-propagation algorithm for 200,000 epochs and then selected the "best" model with the best prediction performance (AUROC) in the validation set. After that, we calculated the AUROC score for the separate test dataset, which was regarded as the final prediction performance in this cross-validation fold. We also used the *dropout* technique [11] with the dropout probability 0.2 for the input layer and 0.5 for other layers.

To avoid confusion, here we further emphasize the subtle difference between the

[1] `deepnet` website: `https://github.com/nitishsrivastava/deepnet`

pretraining and fine-tuning (i.e., error back-propagation) processes in the standard deep learning framework. In the pretraining process, the whole network is regarded as a DBN, i.e., a hybrid (with both directed and undirected edges) generative model in which the elementary modules are restricted Boltzmann machines (RBMs). In both RBM and DBN models, all the vertices in both visible and hidden layers represent binary random variables, which is different from a neural network. Equation 2 in the main paper is the definition of the energy function for an RBM, in which vertices only take binary values $\{0, 1\}$. In fact, the neural network perspective is only taken in the fine-tuning process (i.e., error back-propagation), and the net parameters are initialized based on the pretraining. Here, the network is a (deterministic) feedforward neural net with the same structure as the aforementioned (probabilistic) generative DBN. However, as in the conventional neural networks, all the vertices are now treated as computational units calculating the sigmoid functions and outputting real values in $[0, 1]$.

To show that our prediction model did not suffer from the potential *overfitting* problem, we first claim that the layer-by-layer unsupervised training manner can alleviate the issue of learning a large number of parameters in the current deep learning framework, since the parameters of individual layers during the layer-wise training process actually depend on each other and a certain amount of training data may be sufficient enough to learn accurate parameters in each layer. In addition, it has been well accepted that several techniques and empirical rules developed in the deep learning literature [12] are able to overcome the overfitting problem and yield excellent prediction results in numerous applications [12, 13, 14]. Those default settings of hyperparameters in `deepnet`, such as the coefficient of the L_2 penalty term and dropout probability, that we used in our deep learning framework, generally follow the commonly-used rules. It is true that using different hyperparameter settings for modeling distinct RBPs might produce better prediction performance, but, in practice, it is usually time-consuming and almost infeasible to determine these hyperparameters based on a separate validation set. In fact, the fixed hyperparameter set can also be viewed as part of the prediction model. In our parameter learning process, we only aimed to determine the weight and bias terms in each layer. The above arguments rationalize our hyperparameter settings from a theoretical point of view. To show that our training procedure was also feasible in practice, we also checked the error curves of both training and test data, as shown in Fig. S3. If overfitting occurred in our training process, we expected to observe an increasing error on the test data as the training error steadily decreased. However, such a phenomenon did not happen in our training process. As shown in Fig. S3, the curves of both training and test errors decreased steadily, which implies that overfitting was not an issue in our framework.

Here, we also provide several implementation details of generating RBP binding motifs that are omitted in the main paper. During the top-layer RBM sampling, we first clamped the label vertex to be one, and conducted the mean-field based Gibbs sampling for 10,000 iterations. The same procedure was conducted when clamping the label to be zero for computing ΔP. To sample the RNA words, we set the sampling threshold δ according to the average RNA word probability, i.e., $\delta = 2.5 \times 10^{-4}$ for the base sequence profiles and $\delta = 5 \times 10^{-4}$ for the secondary structural profiles. We sampled 10,000 RNA words and used `WebLogo` [15] to visualize both RNA primary sequence and secondary structural motifs of PTB binding.

Table S2: The AUROC scores of different RNA word lengths for ALKBH5, in which d_1 and d_2 stand for the word lengths for the RNA primary sequence and secondary structural profiles, respectively.

d_1	d_2	AUROC
5	7	0.706
6	8	0.714
7	9	0.701

Table S3: The consistency between the derived RNA tertiary structural motifs and the corresponding primary sequence and secondary structural features for PTB binding. Since a vertex annotation in the 2D diagram of an RNA 3D motif may represent different bases, we calculated the percentage of `C` and `U` in the "best" case for each motif. The five RNA 3D motifs shown here were top predictions from our model. The meanings of vertex and geometric edge annotations in the 2D diagrams of the RNA 3D motifs can be found in [4, 5].

Illustration info	Tertiary structural motifs				
Motif ID	HL_55718.1	HL_86115.1	HL_30008.1	HL_99584.1	HL_99779.1
Loop type	Hairpin loop	Hairpin loop	Hairpin loop	Hairpin loop	Hairpin loop
`CU` percentage	100%	100%	80%	75%	60%
2D diagram	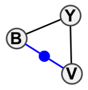	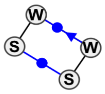	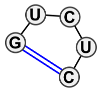	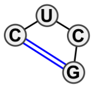	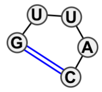

S5 The AUROC scores of different RNA word lengths for ALKBH5

Table S2 summarizes the AUROC scores of different RNA word lengths for ALKBH5, which shows that the slight change of the RNA word length did not have much influence on the prediction performance of our deep learning framework.

S6 The consistency between different types of derived binding motifs for PTB

Table S3 shows the consistency results for the top five 3D binding motifs of PTB derived by our method. The consistency between these RNA 3D motifs and the corresponding base sequence and secondary structural features can be confirmed by the following observations: (i) The fraction of `C` and `U` bases in the top five motifs was high ($\geq 60\%$, see Table S3), which was in line with the fact that PTB has the `CU`-rich sequence binding motif [16] (see also Table 2 in the main paper). (ii) All these top five 3D motifs had hair-

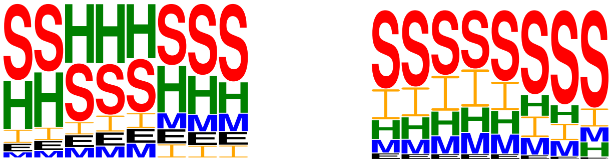

(a) Before inactivation (b) After inactivation

Figure S4: Comparison of the derived secondary structural motifs for PTB binding before and after inactivating the secondary and tertiary structural profiles for 20% of the training samples.

pin loops, which was consistent with the PTB 2D binding motif (i.e., hairpin-loop-rich) derived by our model (see also Table 2 in the main paper).

S7 Results of RBP binding motif generation after partially inactivating structural profiles

To investigate how the encoded RNA structural profiles affect the extraction of structural features in our deep learning framework, we introduced a fraction of unstructured RNA sequences (i.e., bound and unbound sites devoid of structural features) into the training data, and then checked whether our approach can still capture the structural binding motifs. In particular, we randomly selected 20% of positive (bound) and negative (unbound) samples from the training dataset for PTB, and inactivated their secondary and tertiary structural features by constructing the structural profiles randomly. The remaining 80% training data were kept unchanged. Then we performed the same motif generation process as described in the main paper.

The test result showed that the derived secondary structural motif (see Fig. S4(b)) after inactivating the structural features for 20% of the training samples was less notable than the original one (see Fig. S4(a)) derived before inactivation. However, the loops (e.g., multi-, internal and hairpin loops) still held certain conservation in this motif, which was in line with known experimental findings [17]. On the other hand, following the same routine of identifying the tertiary binding motifs as described in our main paper, we found that the distribution difference between $P(3D|bound)$ and $P(3D|unbound)$ became almost negligible (< 0.05), which implies that our framework discovered few dominant 3D structural preferences for PTB binding in this case. In a word, by inactivating the input structural profiles for 20% of the training samples, our approach was not able to fully discover the structural preference of PTB binding, but still captured certain secondary structural binding features.

S8 Supplementary results of the docking experiment

Table S4 presents the docking efficiency of the top five predicted RNA 3D motifs to the RRM domains of PTB except RRM4. Table S5 shows the ratio of `C` and `U` bases among the nucleotides involved in the interactions with PTB.

Table S4: Docking efficiency of the top five predicted RNA 3D motifs to the RRM domains of PTB except RRM4. The docked structures were checked manually, and only those structures that were similar to the corresponding NMR structures and had no serious steric clash between atoms were kept for the calculation of docking efficiency.

Motif ID	RRM1	RRM2	RRM3
HL_55718	0.231	0.341	0.212
HL_86115	0.236	0.301	–
HL_30008	0.208	–	–
HL_99779	–	0.234	0.257
HL_99584	–	–	–
positive	0.216	0.286	0.279

Table S5: The ratio of `C` and `U` bases among the nucleotides involved in the interactions with PTB.

Motif ID	RRM1	RRM2	RRM3
HL_55718	66.7	100.0	100.0
HL_86115	80.0	100.0	–
HL_30008	75.0	–	–
HL_99779	–	80.0	75.0
HL_99584	–	–	–

S9 Supplementary details and results on the PTB binding site prediction in the IRES regions

To predict possible PTB binding sites in IRES, we scanned 40 available IRES regions with window size 26 (which is the average target size of PTB) and step size one, which yielded 15,664 scanned subsequences/windows. Each scanned subsequence was extended by 150 nucleotides in both directions (i.e., upward and downward) for RNA secondary structure prediction. These IRES subsequences were then inputted into both `GraphProt` and our deep learning framework to predict new candidate PTB binding sites. In `GraphProt`, the subsequences with the SVM margins above zero (which was the default parameter setting in `GraphProt`) were treated as binding sites, while in our method, those with output probabilities above 0.6 (which was the best threshold determined by the 10-fold cross-validation procedure) were regarded as PTB targets.

In addition to the overall performance evaluation for all 40 IRES regions, we also checked the prediction results in those 13 IRES regions in which the PTB binding has been validated experimentally in the literature, including *APAF1* [19, 20], *BAG1* [21, 22], *BIP* [23], *MYC* family (*C-MYC*, *L-MYC* and *N-MYC*) [24, 25], *MNT* [26], *MTG8A* [26], *MYB* [26], *P27* [27], *P53* [28, 29], *UNR* [30, 31] and *P58* [32]. As shown in Fig. S5(a),

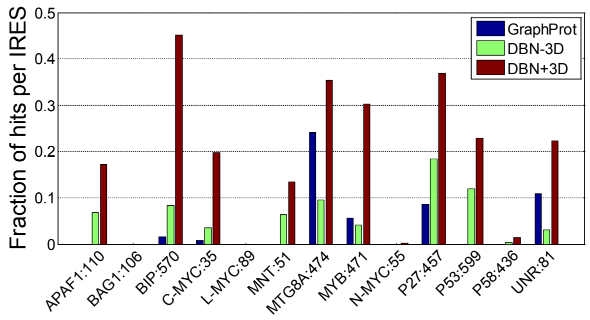

(a) Prediction results in 13 IRES regions with experimental verification of PTB binding

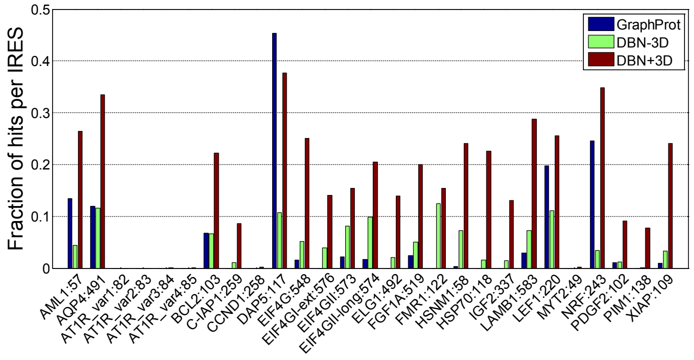

(b) Prediction results in 27 IRES regions without experimental verification of PTB binding

Figure S5: Comparison results on predicting the PTB binding sites of individual IRES regions. (a) Prediction results of different approaches in 13 IRES regions with experimental verification of PTB binding. The x-axis label "a:b" denotes the combination of IRES name "a" and IRESite ID "b" [18]. (b) Prediction results of different approaches in 27 IRES regions without experimental verification of PTB binding. The fraction of hits was defined as the percentage of the predicted bound subsequences among all scanned ones. *DBN+3D* and *DBN-3D* represent the DBN models with and without RNA 3D structural profiles, respectively.

compared to `GraphProt`, the DBN model with RNA 3D structural profiles predicted more binding sites for almost all 13 experimentally-verified IRES regions, except *BAG1* and *L-MYC*, in which all three approaches did not detect any PTB target. In particular, unlike `GraphProt` which failed to identify any PTB binding site for *APAF1*, *P53*, *MNT* and *P58*, our framework succeeded in detecting the PTB-IRES interactions that agreed with the known experimental studies for these IRES regions. In summary, for these 13 IRES, our method captured 84.6% of PTB-IRES interactions that have been verified experimentally, while `GraphProt` only detected 46.2% of them. Moreover, our method identified a number of novel candidate PTB binding targets of the other 27 IRES regions in which the PTB binding has not been reported in the literature (to our best knowledge), as shown in Fig. S5(b). These new predicted binding sites may shed light on the exploration of new interactions between PTB and IRES, and thus provide useful hints for studying the corresponding biological functions.

In the negative control test, we first randomly chose another set of 40 mRNA subsequences (one for each IRES) that did not overlap any of the 40 IRES segments. These 40 mRNA subsequences were also regarded as false or unbound examples to constitute the negative control dataset. In addition, we let the negative control dataset have the same length distribution as that of the IRES set by requiring the length of each unbound sequence to be equal to that of its IRES counterpart. Note that a similar strategy has been used in both `GraphProt` and our DBN model to generate the unbound examples in the cross-validation process.

In addition to *APAF1*, we conducted an additional case study on another IRES *UNR* (upstream of N-ras), which is an internal initiation *trans*-acting factor that regulates mRNA stability and cap-independent translation [31]. *UNR* has been found to contain two PTB binding regions [31], which were denoted as B1 and B2 sites, respectively (see Fig. S7(a)). As in *APAF1*, we also divided the whole *UNR* segment into different regions, i.e., B1, B2 and unbound regions. As shown in Fig. S7(b), although we observed that all three models can detect a number of PTB-*UNR* interactions in both B1 and B2 regions, our prediction model integrating RNA 3D structural information yielded much more consistent results in those PTB binding regions experimentally verified. The false positive ratios of the three models, i.e., `GraphProt`, DBN-3D and DBN+3D, were quite small (0.020, 0.004 and 0.071, respectively), which implies that the predictions in *UNR* using these prediction models (including our deep learning model) did not suffer from the false positive issue.

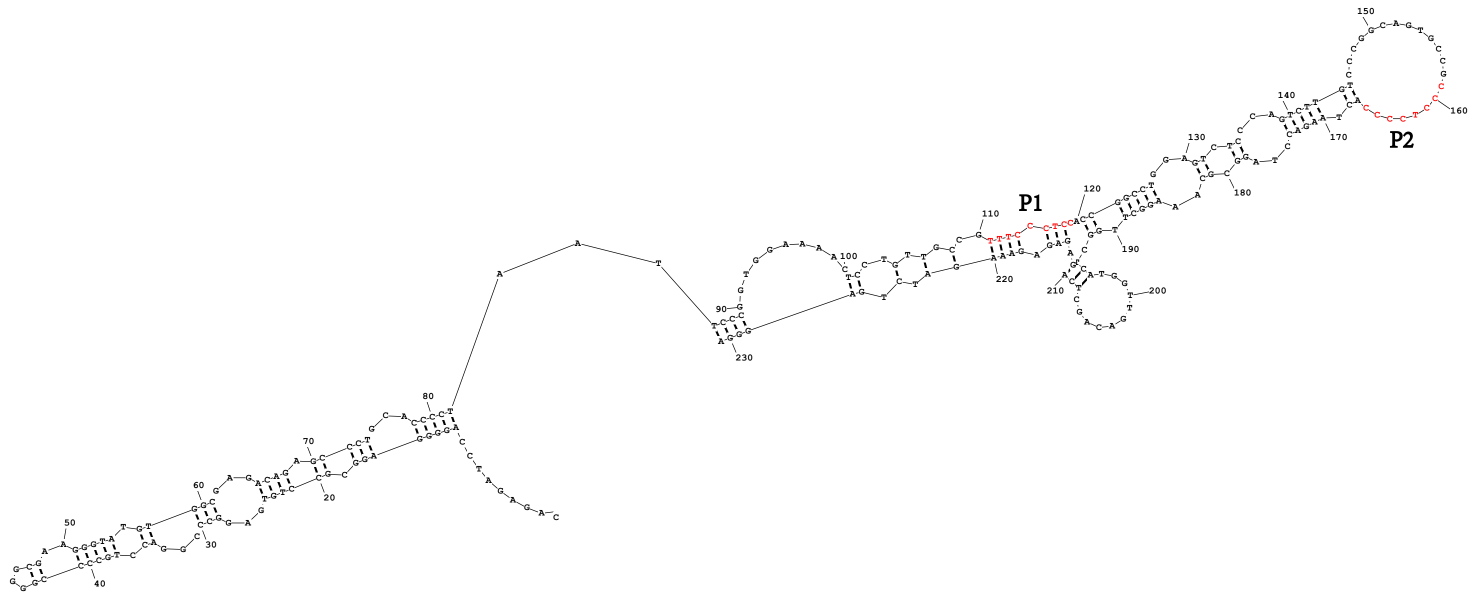

Figure S6: The complete secondary structure of IRES *APAF1*, in which the PTB binding sites, including the P1 and P2 sites (shown in red), have been verified experimentally [20]. The figure was generated using `RNAstructure` [33].

```
36   TGCTGCTTAT GGCGGCGCTG GAGAGGGGGC GCTGAGCTGT TGGGTATGAA GTGTAACAGA
96   ACAGACTTTA CCACCTGAAA CTGCTGCTTC AAGTTCAGAT CAGGCAAGGA ACAAACCTCG
156  TAACAACTAA CAAGACCAAA GAAGAGTACA CTTAAGTTGA AGACACAACA CTTGATCTGA
216  AACAAGAAGT TTGTGCCTAC TCAACAGCTT TGAAAGAGCA CTTCCCAACG CTGCTAGTAG
276  T(CTTTGTTTT CTTC)AGTGCT GTACTGTGAG ATTGCCCGGT ACAGCAGCAG TTGTATTCTT
336  TATTAGCTTG GTAGATCA{TT TTCTCTCGCT CTTTTTTTT}A ATACTAGCAA CTTTCATCCT
396  TTGAAACGTG TGCTGAAAAA GAAGAATCAG CAAATACTAC ATATTTGAGT ATCACTGCG
```

(a) Sequence of *UNR*

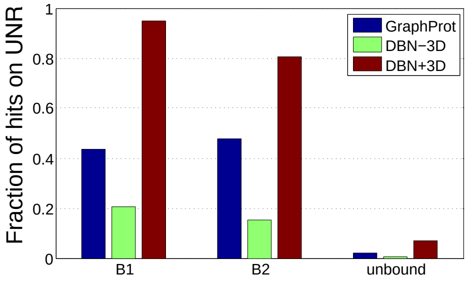

(b) Prediction results of *UNR*

Figure S7: Results on predicting the PTB binding sites of *UNR*. (a) Sequence of *UNR*. Since there is no experimentally-derived structure of *UNR* in the literature (to our knowledge), here we only present its sequence. The number at the beginning of each row denotes the position of the beginning site with respect to the mRNA containing *UNR*. The B1 and B2 sites are the experimentally-verified PTB binding sites [31], which are both marked in red and denoted between round brackets and braces, respectively. (b) Prediction results of *UNR*. The first two x-axis labels represent the prediction results in the B1 and B2 regions, which contained the RNA subsequences in the scanning windows overlapping the B1 and B2 sites, respectively. The last x-axis label represents the prediction results in the *unbound* region, which contained the remaining sites that were excluded from the above regions. The faction of hits was defined as the ratio of the number of predicted bound windows vs. the total number of scanning windows in the corresponding region. *DBN+3D* and *DBN-3D* represent the DBN models with and without RNA 3D structural profiles, respectively.

References

[1] G. E. Hinton and R. Salakhutdinov, "Replicated softmax: an undirected topic model," in *Advances in Neural Information Processing Systems 22*, 2009, pp. 1607–1614.

[2] M. Kline, *Mathematical Thought from Ancient to Modern Times.* OUP USA, 1990.

[3] A. I. Petrov, C. L. Zirbel *et al.*, "Automated classification of RNA 3D motifs and the RNA 3D Motif Atlas," *RNA*, vol. 19, pp. 1–14, 2013.

[4] N. B. Leontis and E. Westhof, "Geometric nomenclature and classification of RNA base pairs," *RNA*, vol. 7, no. 4, pp. 499–512, 2001.

[5] N. B. Leontis, J. Stombaugh *et al.*, "The non-WatsonCrick base pairs and their associated isostericity matrices," *Nucleic Acids Research*, vol. 30, no. 16, pp. 3497–3531, 2002.

[6] S. F. Altschul, W. Gish *et al.*, "Basic local alignment search tool," *Journal of Molecular Biology*, vol. 215, no. 3, pp. 403–410, 1990.

[7] G. E. Hinton, S. Osindero *et al.*, “A fast learning algorithm for deep belief nets,” *Neural Computation*, vol. 18, no. 7, pp. 1527–1554, 2006.

[8] C. M. Bishop, *Pattern Recognition and Machine Learning*, 1st ed., M. Jordan, B. Schölkopf, and J. Kleinberg, Eds. Springer-Verlag New York, 2006.

[9] G. E. Hinton, “Training products of experts by minimizing contrastive divergence,” *Neural Computation*, vol. 14, no. 8, pp. 1771–1800, 2002.

[10] G. Hinton, “A practical guide to training restricted Boltzmann machines,” Dept. of Computer Science, Univ.of Toronto, Tech. Rep. UTML-TR-2010-003, 2010.

[11] G. E. Hinton, N. Srivastava *et al.*, “Improving neural networks by preventing co-adaptation of feature detectors,” *CoRR*, vol. abs/1207.0580, 2012.

[12] Y. Bengio, “Practical recommendations for gradient-based training of deep architectures,” *CoRR*, vol. abs/1206.5533, 2012.

[13] Y. Bengio, A. Courville *et al.*, “Representation learning: a review and new perspectives,” *IEEE Transactions on Pattern Analysis and Machine Intelligence*, vol. 35, no. 8, pp. 1798–1828, 2013.

[14] Y. Bengio, “Learning deep architectures for AI,” *Foundations and Trends in Machine Learning*, vol. 2, no. 1, pp. 1–127, 2009.

[15] G. E. Crooks, G. Hon *et al.*, “WebLogo: a sequence logo generator,” *Genome Research*, vol. 14, no. 6, pp. 1188–1190, 2004.

[16] D. C. Reid, B. L. Chang *et al.*, “Next-generation SELEX identifies sequence and structural determinants of splicing factor binding in human pre-mRNA sequence,” *RNA*, vol. 15, no. 12, pp. 2385–2397, 2009.

[17] S. Auweter and F. Allain, “Structure-function relationships of the polypyrimidine tract binding protein,” *Cellular and Molecular Life Sciences*, vol. 65, no. 4, pp. 516–527, 2008.

[18] M. Mokrejš, T. Mašek *et al.*, “IRESite – a tool for the examination of viral and cellular internal ribosome entry sites,” *Nucleic Acids Research*, 2009.

[19] S. A. Mitchell, E. C. Brown *et al.*, “Protein factor requirements of the Apaf-1 internal ribosome entry segment: roles of polypyrimidine tract binding protein and upstream of N-ras,” *Molecular and Cellular Biology*, vol. 21, no. 10, pp. 3364–3374, 2001.

[20] S. A. Mitchell, K. A. Spriggs *et al.*, “The Apaf-1 internal ribosome entry segment attains the correct structural conformation for function via interactions with PTB and unr,” *Molecular Cell*, vol. 11, no. 3, pp. 757–771, 2003.

[21] B. M. Pickering, S. A. Mitchell *et al.*, “Polypyrimidine tract binding protein and poly r(C) binding protein 1 interact with the BAG-1 IRES and stimulate its activity in vitro and in vivo,” *Nucleic Acids Research*, vol. 31, no. 2, pp. 639–646, 2003.

[22] ——, “Bag-1 internal ribosome entry segment activity is promoted by structural changes mediated by poly(rC) binding protein 1 and recruitment of polypyrimidine tract binding protein 1,” *Molecular and Cellular Biology*, vol. 24, no. 12, pp. 5595–5605, 2004.

[23] Y. K. Kim, B. Hahm *et al.*, “Polypyrimidine tract-binding protein inhibits translation of bip mRNA,” *Journal of Molecular Biology*, vol. 304, no. 2, pp. 119–133, 2000.

[24] L. C. Cobbold, K. A. Spriggs *et al.*, “Identification of internal ribosome entry segment (IRES)-trans-acting factors for the Myc family of IRESs,” *Molecular and Cellular Biology*, vol. 28, no. 1, pp. 40–49, 2008.

[25] L. C. Cobbold, L. A. Wilson *et al.*, “Upregulated c-Myc expression in multiple myeloma by internal ribosome entry results from increased interactions with and expression of PTB-1 and YB-1,” *Oncogene*, vol. 29, no. 19, pp. 2884–2891, 2010.

[26] S. A. Mitchell, K. A. Spriggs *et al.*, “Identification of a motif that mediates polypyrimidine tract-binding protein-dependent internal ribosome entry,” *Genes & Development*, vol. 19, no. 13, pp. 1556–1571, 2005.

[27] S. Cho, J. H. Kim *et al.*, “Polypyrimidine tract-binding protein enhances the internal ribosomal entry site-dependent translation of p27Kip1 mRNA and modulates transition from G1 to S phase,” *Molecular and Cellular Biology*, vol. 25, no. 4, pp. 1283–1297, 2005.

[28] R. Grover, P. S. Ray *et al.*, “Polypyrimidine tract binding protein regulates IRES-mediated translation of p53 isoforms,” *Cell Cycle*, vol. 7, no. 14, pp. 2189–2198, 2008.

[29] R. Grover, A. Sharathchandra *et al.*, “Effect of mutations on the p53 IRES RNA structure: implications for de-regulation of the synthesis of p53 isoforms,” *RNA Biology*, vol. 8, no. 1, pp. 132–142, 2011.

[30] B. Schepens, S. A. Tinton *et al.*, “A role for hnRNP C1/C2 and unr in internal initiation of translation during mitosis,” *The EMBO Journal*, vol. 26, no. 1, pp. 158–169, 2006.

[31] S. Cornelis, S. A. Tinton *et al.*, “UNR translation can be driven by an IRES element that is negatively regulated by polypyrimidine tract binding protein,” *Nucleic Acids Research*, vol. 33, no. 10, pp. 3095–3108, 2005.

[32] S. Ohno, M. Shibayama *et al.*, “Polypyrimidine tract-binding protein regulates the cell cycle through IRES-dependent translation of CDK11p58 in mouse embryonic stem cells,” *Cell Cycle*, vol. 10, no. 21, pp. 3706–3713, 2011.

[33] J. S. Reuter and D. H. Mathews, “RNAstructure: software for RNA secondary structure prediction and analysis,” *BMC Bioinformatics*, vol. 11, p. 129, 2010.
